# 35,000 years of recurrent visits inside Nerja cave (Andalusia, Spain) based on charcoals and soot micro-layers analyses

**DOI:** 10.1038/s41598-023-32544-1

**Published:** 2023-04-11

**Authors:** Mª Ángeles Medina-Alcaide, Ségolène Vandevelde, Anita Quiles, Edwige Pons-Branchu, Iñaki Intxaurbe, José Luis Sanchidrián, Hélène Valladas, Damien Deldicque, Catherine Ferrier, Eva Rodríguez, Diego Garate

**Affiliations:** 1 Université de Bordeaux, UMR CNRS 5199 PACEA, Bâtiment B2 Allée Geoffroy Saint Hilaire, 33600 Pessac, France; 2 Universidad de Córdoba, HUM-781, 14071 Córdoba, Spain; 3 Laboratoire des Sciences du Climat et de L’Environnement, LSCE/IPSL, CEA-CNRS-UVSQ, Université Paris-Saclay, 91191 Gif-sur-Yvette, France; 4 Institut Français d’Archéologie Orientale, Pôle Archéométrie, Cairo, Egypt; 5 Departamento de Geología, Euskal Herriko Unibertsitatea/Universidad del País Vasco, 48940 Leioa, Spain; 6 Laboratoire de Géologie, Département de Géosciences, École Normale Supérieure, CNRS, UMR 8538, PSL University, Paris, France; 7 Instituto Internacional de Investigaciones Prehistóricas de Cantabria (IIIPC), Universidad de Cantabria, 39005 Santander, Spain

**Keywords:** Anthropology, Archaeology

## Abstract

Charcoal and micro-layers of soot trapped in speleothems from the inner galleries of Nerja Cave were analysed through an interdisciplinary study. The absolute dating of the prehistoric subterranean activity of the cave and the identification of different phases of visits to the deep parts are presented and discussed. The charcoal analysis includes anthracological analysis and SEM–EDX. The soot analysis includes optical microscopy, Raman spectroscopy and TEM–EDX, and the microcounting of soot microlayers. The ^14^C dating of 53 charcoals identified 12 phases of prehistoric visits to the cave between 41,218 and 3299 cal. BP, putting back the origin of human occupation of this emblematic cave by 10,000 years. The interdisciplinary analysis of the soot microlayers allowed us to perform a high-precision zoom on the last three visitation phases identified by Bayesian analysis (8003–2998 cal. BP.), demonstrating that these phases contain at least 64 distinct incursions, with an average of one visit every 35 years for the Neolithic period. Spatial analysis showed that not all areas of the cave were used in the same periods, highlighting the repetition of visits to certain specific sectors of the Lower Galleries of the cave. Lastly, the anthracological data indicate a cross-cultural and unique use of *Pinus* tp. *sylvestris*-*nigra* wood for lighting activities over an extended period between the Gravettian and Upper Magdalenian.

## Introduction

Deep karst visits during the Paleolithic are known by remains such as rock art, fire remains or even human constructions such as the one found at Bruniquel cave^1^. It is however difficult to determine if these remains were left by single visits or recurrent ones, as they are often found on the soil surface in caves, devoid of a time-stratigraphic context to provide chronological support. Here, by the ^14^C dating of more than 60 samples of lighting and fire remains (charcoals and soot layers) and two abstract rock art representations, we are able to present a robust Bayesian model that constrains the periods of occupation for the internal prehistoric activity in Nerja cave. Using the multi-analytical identification and micro-counting of soot layers trapped in a stalagmite, we also provide the minimum number of visits and their recurrence.

Nerja Cave (Malaga, Andalusia, Spain) is one of the main sites for the study of prehistoric groups in the Western Mediterranean. An extensive archaeological and palaeontological sequence has been recovered in its entrance rooms, including remains from the Gravettian, Solutrean, Magdalenian, Epipalaeolithic, Neolithic and Chalcolithic chrono-cultures. From the discovery of the cave (1959) to the present day, this sedimentary deposit has been examined by different research teams, resulting in extensive scientific and cutting-edge knowledge (including numerous radiocarbon dates) about the prehistoric inhabitants of Southwest Europe^2–6^.

The interior spaces of the cave are structured by a large endokarst volume of more than 4.8 km of topographic development, in which there are numerous slopes and complex speleological passages. Sunlight does not penetrate these areas and artificial lighting is essential for access. These sectors have received less archaeological attention since the discovery of the cave, although they contain a unique wealth of heritage, including one of the most richest and oldest Palaeolithic Art sites in southern Spain^7^.

In recent years, the study of the Internal Archaeological Context (henceforth IAC)^8,9^ has been prioritized, through an intensive revision of the walls and floors of the internal galleries, as well as through the use of new technologies on site (DStretch software, digital portable microscope, etc.) for the detection and preliminary analysis of different remains of prehistoric subterranean occupation, followed by their laboratory analysis. This has enabled a holistic and up-to-date understanding of the different uses and visits to the deep areas of the cave. Likewise, the number of cave motifs found has considerably increased and numerous traces of the different Pleistocene and Holocene visits to the interior of the cave have been discovered. These are linked to subterranean activities of a heterogeneous nature, including the execution of Palaeolithic graphic manifestations^7^, the Palaeolithic modification of the endokarst geomorphology^10^ and the use of the cave for burial purposes during the Recent Prehistoric period^11^.

Most of the archaeological remains found on the floor of the internal areas of Nerja cave are pieces of charcoal from the woody fuel used for lighting the cave during prehistoric times. These residues correspond to the lighting of fires and fixed “lamps” but, above all, to the use of wooden torches^12–15^. As our previous experimental research indicates^16^, the charcoal from the torches is usually found scattered and isolated, with no other combustion residue nearby, the result of its intermittent detachment during human transit carrying this lighting tool (like the breadcrumbs in the story of *Hansel and Gretel*). In this regard, these lighting residues (charcoals from prehistoric lighting systems) are remains from the different prehistoric visits. Their ^14^C dating allows the chronological determination of prehistoric underground activity, as well as knowledge of the unique or multiphasic nature of the prehistoric activity inside the cave.

In this paper we present the first high-precision Bayesian model for the chronological, multi-phase, absolute-scale characterisation of different prehistoric visits to the deep sectors of Nerja cave. The paper includes 68 radiocarbon datings (48 of them unpublished) carried out by two different laboratories, in USA (Beta Analytic) and in France (Laboratoire des Sciences du Climat et de l’Environnement, first on the Tandétron then on Artémis, LMC14). Before their inclusion in the Bayesian model, the dates were subjected to a “validation filter”^3^ based on analytical and physico-chemical criteria, in order to robustify the model. In addition, the results have been linked to other chronometers, in particular, to 7 new ^14^C datings on carbonates from a stalagmite with soot microlayers to provide the model with greater accuracy and resolution. In total, 35,000 years of human occupation in the deep karst and at least 64 different phases have been identified, so far the largest number of visits known for a prehistoric cave in Europe.

## Materials and methods

The materials examined are combustion residues, mainly charcoal pieces and soot microlayers, from different lighting systems used during prehistoric times to enter the deep areas of the cave, as well as some of the tiny charcoal particles from the pigment of black marks located on the cave walls. Charcoal was identified through anthracological analysis including SEM–EDX. Soot microlayers, located inside a small stalagmite collected, were studied through optical microscopy observation and characterized with Raman spectroscopy and TEM–EDX analysis. The ^14^C dates obtained on these residues of charcoal and soot were then subjected to a Bayesian analysis using Oxcal 4.4 (after performing a “validation test" on each sample) to identify the different phases of the visits inside the cave, the transition intervals and the duration of each visit. Finally, the visits of the Holocene phases obtained through Bayesian analysis were refined by micro-counting the numerous layers of soot inside the stalagmite. More detailed information on the methodology followed can be found in the Supplementary Information (S1).

**Figure 1:**
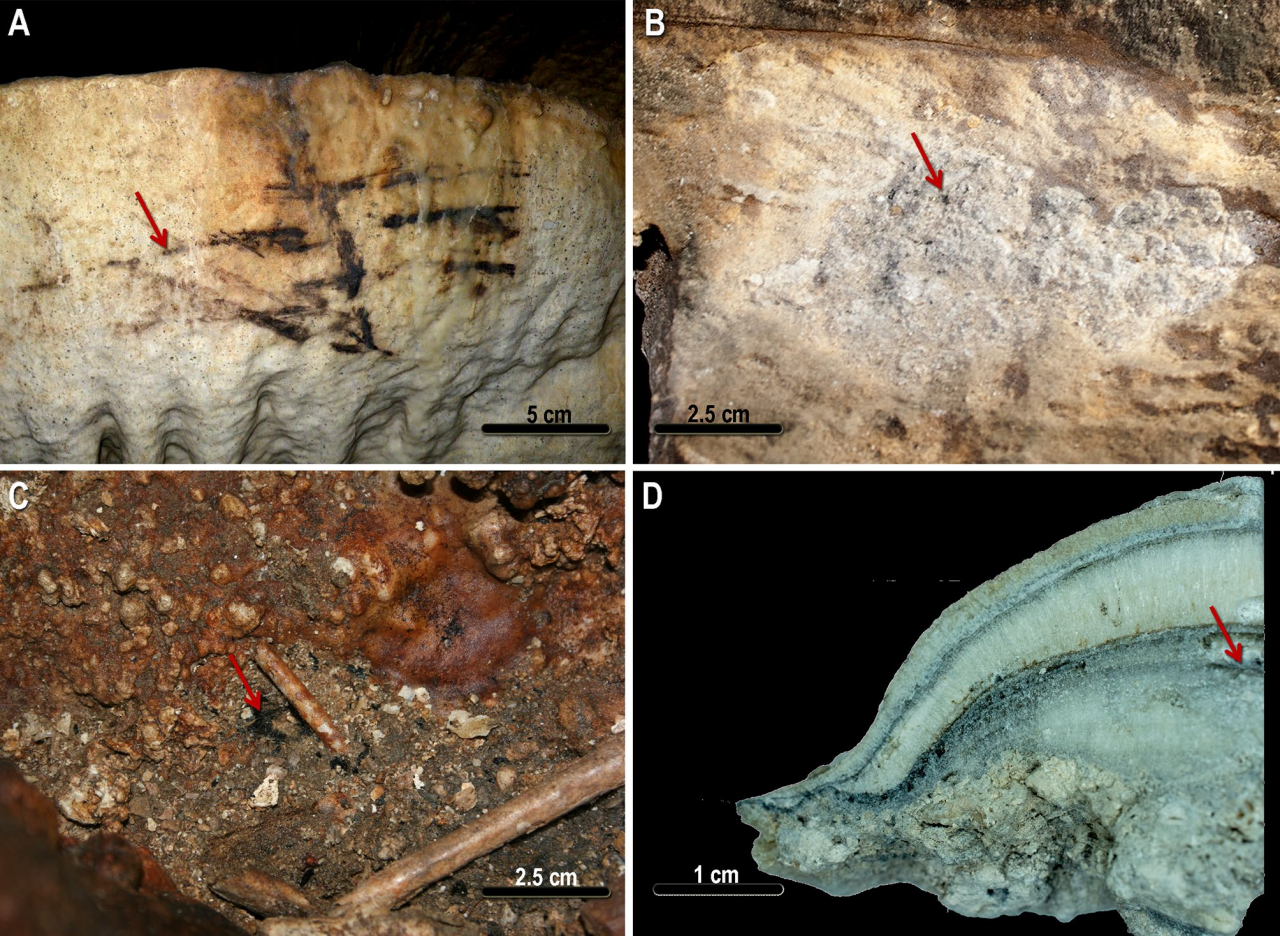
Image composition of the materials. (**A**) Black mark (dating number 33). (**B**) Micro-charcoal inside fixed lamp (dating number 43). (**C**) Scattered charcoals (dating number 54). (**D**) GN16-08 stalagmite section. The red arrows point to one of the samples, analysed both by TEM–EDX and Raman micro-spectroscopy.

Three types of sampling plan were employed depending on the nature and localization of the samples:

- *Samples from the floor* An aseptic collection technique was developed for charcoal lying on the floor, in interstices or cavities at surface or semi-surface level. All rooms with prehistoric human occupation were sampled. The number of samples per room was proportional to the number of charcoals located in each sector, with the aim of obtaining a representative chronological approximation on a global scale. Sampling began in an elevated room, the Cascade Room, in order to avoid dating samples linked to external sedimentary deposits that may have migrated or percolated inside the cave through different taphonomic processes. The tourist remodelling of some areas of the cave was the main limitation for the sampling of some internal sectors. We therefore sampled only residues that had not undergone intense post-depositional movements. Thus, 66 charcoal pieces samples were selected from the floor. Of these samples, four came from the interior of concavities interpreted as possible "lamps" or "fixed combustion points”^12–14^ (Fig. 1B) (numbers 28, 43, 65, 66, see Supplementary information S2), and 3 from the interior of possible fixed fires (numbers 60, 64, 36, Supplementary information S2). Samples 11 and 14 relate to the possible wick of a lamp on a shell^15^. The other samples are scattered charcoal, without the presence of other combustion residues nearby; based on the dispersion of remains from previous experimental activities^16^, we relate these remains to the use of wood torches. Although the remains were generally found at surface level (Fig. 1C), we occasionally made small probes to locate residues a few centimetres deeper, especially for the detection of combustion residues located inside the fixed “lamps”.
- *Samples from black marks* tiny particles (< 2 mm) of charcoal pigment from 2 black marks present on the cave walls were micro-sampled, also with aseptic instrumentation (sample numbers 33, 34, see Supplementary information (S2) (Fig. 1A). These black marks correspond to the numbers 265 and 284 of the cave art catalogue^7^ and their dating was previously published by some of us^17^.
- *Stalagmite with soot microlayers* A 9 × 3.5 cm high stalagmite was collected in the Cataclysm room. For the study of its internal structure, the stalagmite was sectioned into four quarters. To characterize the soot, Raman analysis was performed on several of the black micro-levels present in its internal structure, as well as TEM–EDX analysis of a sample powder extracted by scraping with a scalpel from lower black microlayers of the stalagmite (Fig. 1D).

## Results

The Bayesian model for the internal rooms of Nerja cave, through radiocarbon dating on charcoal, includes 53 results, as 15 of them did not pass the validity analysis. There are several samples that were dated before their identification. Therefore, for these few samples, we cannot confirm that they correspond to organic residues of prehistoric underground activities (samples 1–10, 20–21, 67–68). Other samples were excluded because they were linked to the contemporary use of the cave (samples 1–10), as well as one date that shows a high deviation (sample 64), which could hinder the precise chronological identification of their respective phases.

In Supplementary information (S2) we have included the results of all ^14^C datings on charcoal (not only those implemented in the Bayesian model) and additional information about the nature and the localization of the samples inside the cave. Twenty-seven samples were characterised as “indeterminate charcoal”, 15 as “*Pinus* tp. *sylvestris*-*nigra*”, 1 as “*Pinus* tp. cf. *pinea*-*pinaster*”, 3 as “*Pinus* sp.” and 5 as “conifer”. The predominant characterization as "indeterminate charcoal" is due to the high presence of charcoal with a strongly altered structure in relation to the presence of vitrification, the surface exposure of most of the remains (trampling, hyperfracturing, etc.) and the small size of some samples.

**Figure 2:**
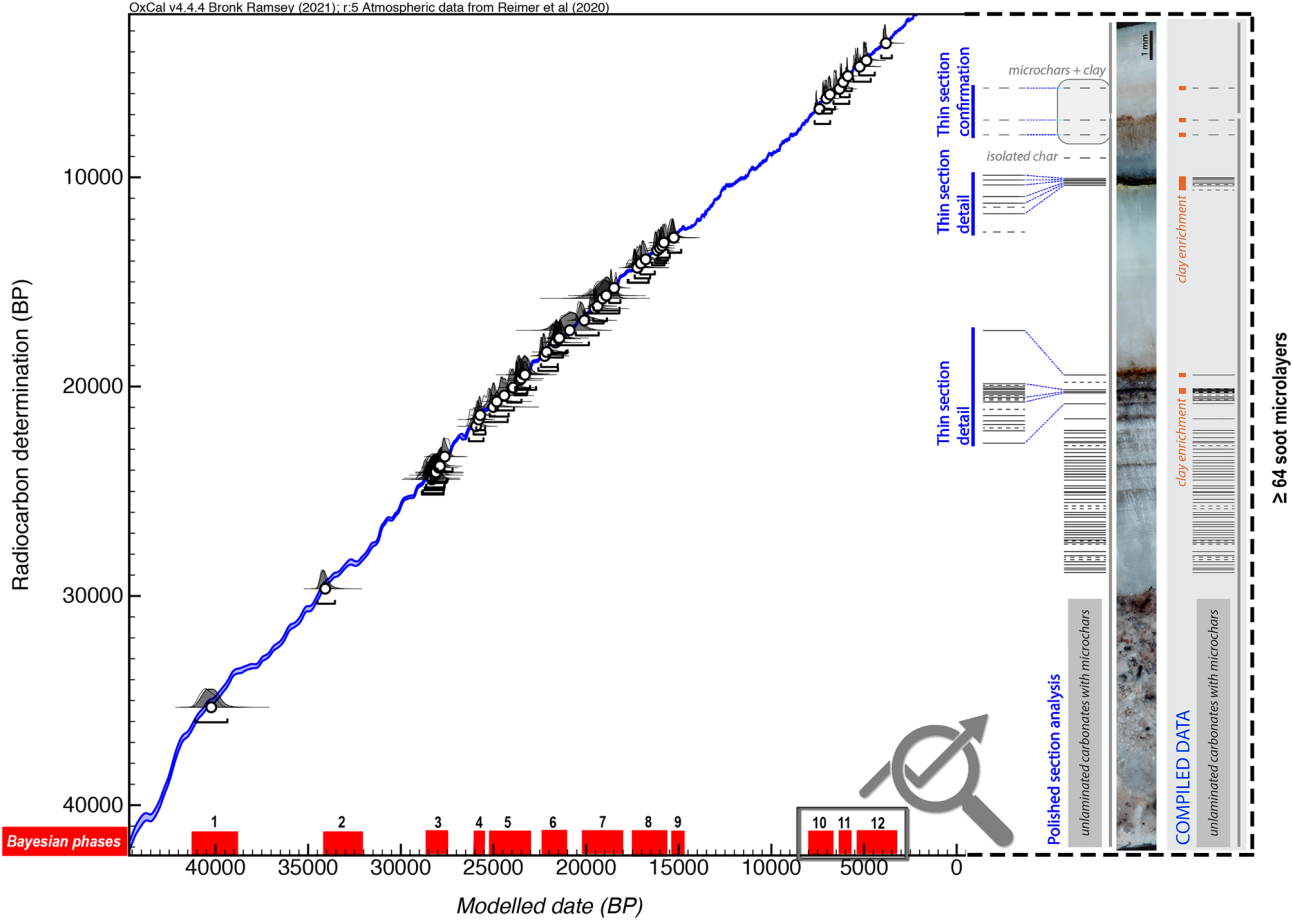
Curve plot with the different phases of visits to the interior of Nerja cave identified by a Bayesian model from charcoal (in red) and image of the different micro-layers of soot and micro-charcoal of the GN16-08 stalagmite, which increase the minimum number of occupations to at least 64 for the last 3 Bayesian phases. The succession of soot films in the carbonates is represented as barcode diagrams. Bars represent soot films and dashed lines represent probable soot films. The long vertical grey line next to the barcode represents speleothem total thickness.

The Bayesian model suggests at least 12 distinct phases of visits to the interior of Nerja cave between 41,218 and 3299 cal BP, with an agreement index (Aoverall) of 98 (see Supplementary information S4–S5 for more information). These phases of visits to the interior of the cave correspond to the specific chronocultural periods for the prehistoric regional context and with transitional periods: Early Aurignacian (phase 1), Recent Aurignacian (phase 2), Gravettian (phase 3), Lower Solutrean (phase 4), Middle Solutrean (phase 5), Upper Solutrean (phase 6), transition between the Upper Solutrean and Lower Magdalenian, and Lower Magdalenian (phase 7), Middle Magdalenian (phase 8), Upper Magdalenian (phase 9), Early Neolithic (phase 10), Recent Neolithic (phase 11) and Copper Age (phase 12). For the start and end dates of each phase see Supplementary information S5, table rows “Boundary Start Phase” and “Boundary End Phase” (Fig. 2).

There are 12 possible transition intervals between the different phases. One of them, separating phases 9–10 (between the Upper Magdalenian and the Early Neolithic), has a notable chronological amplitude (> 6000 years). At the same time, there are 8 transition periods that could correspond to 0 years (almost-continuous phases, taking into account the minimum values), in particular, those between phases 1–2 (both could be included in the Aurignacian, the former probably belonging to the ancient phase and the latter to the evolved stage), 4–5 (between the Lower Solutrean and the Middle Solutrean), 5–6 (between the Solutrean and the Middle Solutrean), 6–7 (around the Upper Solutrean and the transition between the Upper Solutrean and Lower Magdalenian), 7–8 (between the Lower Magdalenian and Middle Magdalenian), 8–9 (between the Middle Magdalenian and Upper Magdalenian), 10–11 (between the Early Neolithic and Recent Neolithic), and 11–12 (between the Recent Neolithic and Chalcolithic). In these quasi-continuous periods the term “phase” is debatable. However, we have taken into account the average values for the identification of transitional periods, which in no case is 0. For Interval Transition Phases see (Supplementary information S5-, table rows “Interval Transition Phases”) (Fig. 2).

The chronology of the soot microlayers found in the GN16-08 stalagmite was determined by the analysis of CaCO_3_ layers deposited before and after them. The soot microlayers at the base of the stalagmite were deposited between 7562 and 6736 cal. BP (0% DCP); the soot microlayers from the upper level of the stalagmite were deposited between 6836 and 2998 cal. years BP assuming 0% of DCP (see Supplementary information S6 for more information).

In the Raman spectra carried out on the soot levels, the D and G bands typical of carbon bounds in polycyclic carbonaceous materials can be recognised, together with a peak at 1085 characteristic of carbonates^18,19^. TEM–EDX observations revealed spherical carbon particles of soot aggregates similar to those found in Domica cave (Slovakia)^20^. In Nerja Cave, similar particles were observed inside a Palaeolithic fixed lamp in the upper galleries^13^, but soot residues were also located in another speleothem fragment inside the cave^21,22^. Microscopic observation revealed that clay deposits were only associated with the microcharcoal or soot levels, suggesting that clay was not brought in by percolation at different times of the stalagmite formation but that the human visits to the cave contributed to the suspension of clays that re-deposited in the stalagmite. Our interdisciplinary analyses (Optical Microscopy, Raman and TEM–EDX) confirm that the black levels observed in stalagmite GN16-08 are the result of particulate emissions from wood combustion, mainly composed of concentric nanoparticles of soot^26^, sometimes including also microcharcoal and occasionally clay re-deposited during human occupations (see Supplementary information S7).

The fuliginochronological analysis^23–25^ of the internal structure of this stalagmite allowed us to identify a minimum of 64 occupations (a Minimum Number of Occupations—MNO that can be increased to 82 if we also count uncertain soot films and microcharcoal alignments. This second case will be included in brackets from now on) (see supplementary information S7 for more information) (Fig. 2). If we relate these data to the phases determined by Bayesian analysis, we can state that at least 58 (71) different occupations occurred in phases 10 and 11 (Early Neolithic and Recent Neolithic) with no apparent hiatus between these two phases (a result that is consistent with the Bayesian model from charcoal), and at least 6 (8) visits in phase 12 (Copper Age).

Based on the duration in years of each phase (Internal duration) obtained by the Bayesian model (Supplementary information S5), and correlating these data with the microcount of the soot microlayers and their respective CaCO_3_ dating, we suggest that between the Early Neolithic and the Recent Neolithic (phases 10 and 11) there was an average of one visit inside the cave every 35 years (if we consider the average chronological values; it is also possible that these Holocene visits occurred continuously over a certain period of time). This level of precision by the study of underground prehistoric activity is pioneering and clearly shows the potential of the applied methodology, and the importance of Nerja cave as a site for the study of the use of caves during prehistoric times.

**Figure 3:**
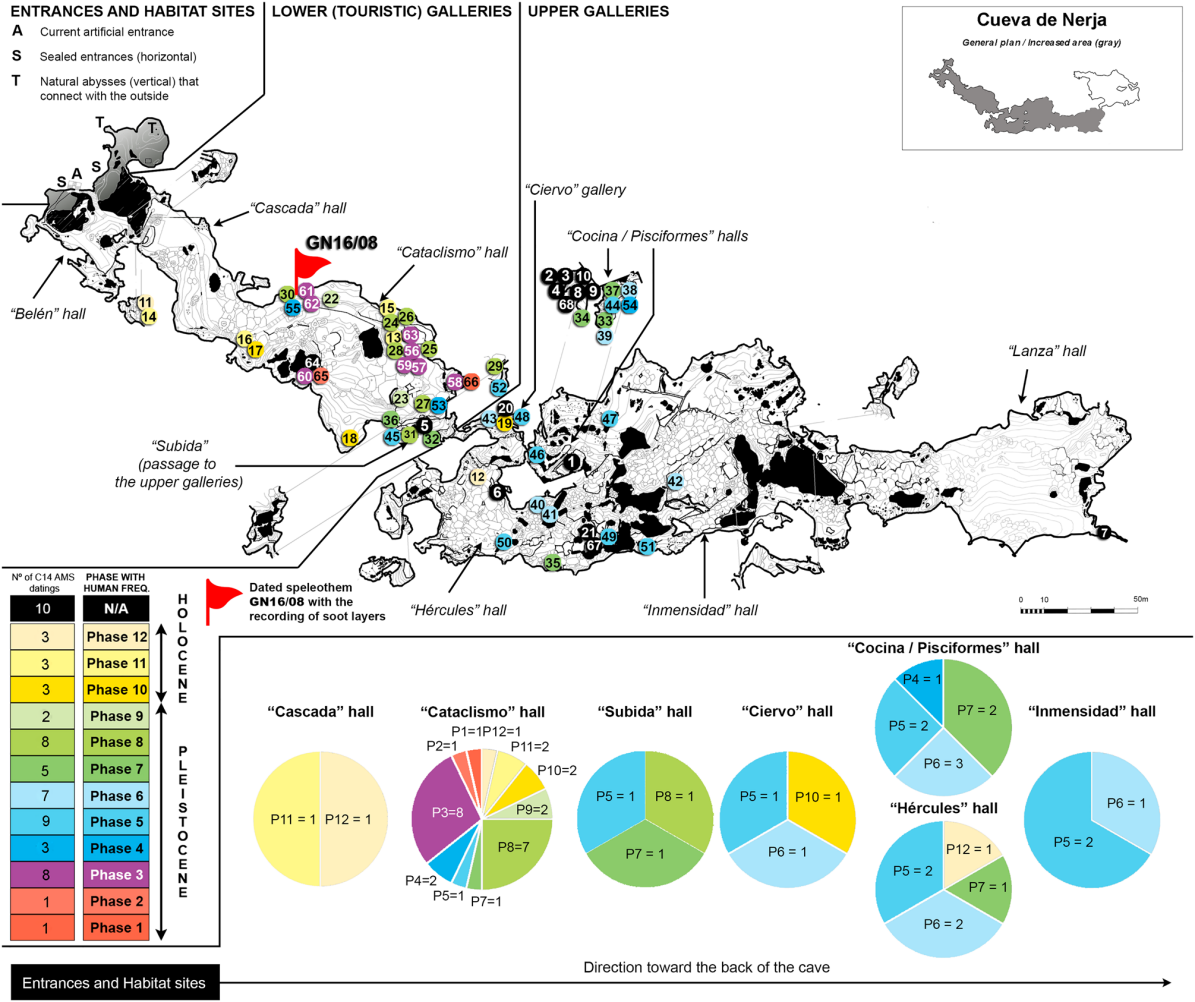
Planimetry of Nerja cave with the location of the analysed samples (modified from Medina-Alcaide 2019^27^). Circles indicate the charcoal samples dated by ^14^C, and the red flag marks the place of the stalagmite GN16-08 with the micro-levels of soot. The colour of the circles corresponds to the Bayesian phases from charcoal dating. In the lower right part of the image, the number of phases is represented by means of circle graphs for each room of the cave. Specific information about each sample can be found in “Supplementary Materials”. The numbers in the circles correspond to the number of samples.

The spatial distribution of the studied samples (Fig. 3) indicates that the Lower Galleries of Nerja cave (Cascade Room and Cataclysm Room) are the sectors with the sectors with the highest number of distinct visits; they evidence 12 phases, between 41,336 to 4604 cal. BP, including the last three phases of the Bayesian model (Early Neolithic, Recent Neolithic and Copper Age) which can be broken down into at least 64 distinct visits according to the soot micro-layers. These are the sectors closest to the present and prehistoric entrances, which are currently prepared for tourist visits. However, without the recent modifications (stairs, walkways, widening of narrow passages, etc.) the topography of these galleries may have been very complicated, with very difficult obstacles (vertical passages, holes, ledges, narrow passages, etc.) and a speleological development of more than 1 km with continuous topographical features. Most of the dated samples from this area were located in various peripheral sectors with Palaeolithic art from the Cataclysm Room, with the exception of two samples from phases 11 (Recent Neolithic) and 12 (Copper Age), which were collected in the Cascade Room, next to a *Pecten* sp. shell that might have functioned as a mobile lamp^15^.

The 64 incursions detected through the fuliginochronological study also correspond to visits to the Lower Galleries of the cave, and in particular to a peripheral and elevated room known as “Plataforma Raspador”, which is a platform of about 47 m^2^ where there are projections of red pigmentation on the west wall. In addition to the 64 phases relating to the Holocene chronology, the Bayesian phases 8–6 (Upper Solutrean, transition between the Upper Solutrean and Lower Magdalenian, Lower Magdalenian, and Middle Magdalenian) have been found in this particular sector of the cave. Therefore, the recurrence of visitation phases can be traced back to specific areas of the cave.

The Upper Galleries of the cave host the deepest areas of the cavity (a distance of between 1 and 4.8 km from the entrance), and are separated from the Lower Galleries by a very difficult speleological obstacle (a vertical wall of more than 30 m that can only be climbed by a steep and dangerous path, with narrow passages and small ascents of 7 m or less). These extremely difficult areas to access were visited since the beginning of Phase 4, from the Lower Solutrean, around 26,188 cal BP. All successive phases of visits up to Recent Prehistoric times are recorded here, with the exception of phases 8–9 relating to the Middle Magdalenian and Upper Magdalenian, respectively. In addition, other dates were obtained in these deep sectors (including chronologies from the Mousterian, as well as the Epipalaeolithic), although they did not pass the validity filter and thus remain, for the time being, unconfirmed phases for the internal occupation of the cave.

Phases 5 and 6, corresponding to the Middle Solutrean and Upper Solutrean periods (25–21,000 cal BP), appear to be the periods in which the anthropization of the cave was the most expansive in terms of spatial extension, ranging from the entrance rooms to the Immensity Room (more than 3 km from the outside). Furthermore, this incursion into the deep underground environment is the oldest known to date. In contrast, the least spatially developed periods of prehistoric visits to the interior of the cave took place in the early (< 3: Aurignacian and Gravettian) and late Upper Palaeolithic (8–9: Middle and Upper Magdalenian) phases, which are limited to the Lower Galleries (Cataclysm Room).

## Discussion

Currently, Nerja Cave is the Palaeolithic Art cave in Europe where the greatest number of phases of distinct prehistoric visits to internal areas have been recorded, making it a unique and exceptional place for understanding the recurrent use of the subterranean environment during prehistoric times.

The Bayesian analysis, including ^14^C dating from charcoal, confirmed the hypothesis of the existence of at least 12 distinct visitation phases between the Aurignacian and the Copper Age (41,218–3299 cal BP). The study of the soot levels present in a Holocene stalagmite made it possible to reach a higher degree of precision for the last three phases, relating to the Early Neolithic, Middle-Final Neolithic and Copper Age (8003–3299 cal BP), increasing the minimum number of incursions to 64 for this period. In total, at least 73 phases of distinct visits to the interior of the cave between the Upper Palaeolithic and Recent Prehistory have been recorded.

Furthermore, this work extends the origin of the prehistoric occupation of the Nerja cave by 10,000 years. The archaeological and chronometric analysis of the sedimentary deposits present in the entrance areas of the cave confirmed an extensive period of use of the cave between 30,000 and 4000 years, encompassing the Gravettian, Lower Solutrean, Middle-Upper Solutrean, Upper Magdalenian, Epipalaeolithic, Mesolithic, Early Neolithic, Middle-Recent Neolithic and Chalcolithic chronocultures^2–6^. Our study of the internal areas of the cave allowed us to extend the periods of occupation recorded in the cave, identifying for the first time two phases of visits relating to the Aurignacian period, namely phases 1 and 2 (Early Aurignacian and Recent Aurignacian), the former corresponding to the Early Aurignacian and the latter to the Evolved Aurignacian.

The oldest date included in the Bayesian analysis (dating 66) is difficult to classify in a specific technocomplex, since in the stratigraphic sequences of the external rooms of the cave there are no such old dates, and at the regional level there is no consensus for the end of the Middle Paleolithic and the beginning of the Upper Paleolithic^28–32^. Therefore, and with the data available today, we could relate this date to some transitional moment between the Middle and Upper Paleolithic. As for the dating 65, this could fit with the recent Aurignacian. This is a period that has already been detected in the east of the Iberian Peninsula with occupations that include the characteristic industry and personal ornamentation^33–39^. This second dating would be one of the few evidences of recent Aurignacian in the south of the Iberian Peninsula, coeval with the recently published dates obtained in a sector near the prehistoric entrance of the Ardales cave^40^ (Supplementary information S8).

Cross dating (^14^C and U/Th) of CaCO_3_ layers covering red dots established that these pictorial elements could be made before 25,370–25,000 cal BP (*terminus ante quem*)^41^, that is, at some time between phases 1–5 defined by the Bayesian model (Early Aurignacian -phase 1-, Recent Aurignacian -phase 2-, Gravettian -phase 3-, Lower Solutrean -phase 4- and Middle Solutrean -phase 5). Thus, at least part of the red-coloured paleolithic art found in the Lower Galleries, mostly composed of simple signs (such as dots, and probably also, paired lines, lines…) could be made between these first occupation phases.

Likewise, certain palaeolithic figures that accompany these simple signs in the Lower Galleries of the cave are thought to correspond to this pre-Magdalenian chronology from a chronostylistic point of view. For example, the figures of hinds in these galleries that have a trilinear convention and/or an exaggerated projection of the necks of some animals link better with phases 3–4 (Gravettian, Lower Solutrean and Middle Solutrean). Furthermore, the horses with the “duck–beak” convention present in this part of the cave could be linked to phase 4 (Middle Solutrean), based on the direct dating of a similar motif in La Pileta cave (Andalusia, Spain)^42^ and the preferential location of these motifs on the plaquettes from this period in the Parpalló cave (Valencia, Spain)^43^.

The occupation phases 6–7 (Upper Solutrean—transition between the Upper Solutrean and Lower Magdalenian), could be related to motifs ascribed to the Advanced Solutrian from a stylistic and formal perspective^7^. Likewise, occupation phase 7 (Lower Magdalenian) could be related to the execution of the black marks; in fact, of the five dates that are grouped together in this period, two correspond to direct dating dates of this type of parietal marks^17^.

Additionally, for the first time in Nerja Cave, the presence of visits that can be chronologically placed in the Lower Magdalenian (phase 7) and Middle Magdalenian (phase 8) has been confirmed. In the sedimentary sequences of the entrance rooms of this cave, this episode coincides with an erosive process that probably dismantled part of the archaeological sequence, explaining its absence^2,5^. Thus, we suggest that the new dates presented in this work, relating to the Lower Magdalenian period, support a continuity of occupation in Nerja cave, and therefore in the south of the Iberian Peninsula, between the Solutrian and Magdalenian periods. In this respect, it is worth highlighting the suitability of the chronological study of the Internal Archaeological Context to strengthen and extend the sequence of use of the caves, as the archaeological remains are generally located beyond the reach of erosive processes that most often take place in the external areas of caves.

Most of the radiocarbon datings presented in this work are related to the different visits to the interior of the cave. Only dates n.33 and n.34 correspond to direct results from the pigment of certain palaeolithic motifs, in particular non-figurative black marks (phase 7-Lower Magdalenian). Therefore, the results of this work alone cannot be used to date the Palaeolithic Art of the interior of Nerja cave. However, we consider that these data can be used to build arguments about the chronology of the different uses of the cave (only one of them being the execution of the graphic manifestations), together with other chronometric, archaeological and/or stylistic data. For example, the absence of carbons of Aurignacian, Gravettian or Middle and Upper Magdalenian chronology in the Upper Galleries of the cave suggests that most probably the visits to these areas (including the one(s) linked to the artistic execution) do not correspond to these periods, and that therefore, the pictorial activity at this site could be related to some time(s) between the Early Solutrian and the Lower Magdalenian, as suggested by the indirect (and direct, in the case of the Lower Magdalenian) dating obtained in this area. In this argument, we must also consider that charcoal from these phases may not have been found, for example, due to taphonomic processes, because the activity was limited in these areas or that the remains may have been destroyed by later occupations.

The Occupation phase 9 (Upper Magdalenian) does not seem to be linked to the execution of Palaeolithic art inside the cave, at least according to the chrono-stylistic data currently available for the cave's rock art. Following some chrono-stylistic parallels, the pisciform pictures of the Upper Galleries have been attributed to the Upper Magdalenian^7^, but the chronocultural ascription of this pictorial group is controversial and is still an open question today^22,44^. For the Upper Magdalenian, although we did not find clear evidence of rock art in the internal rooms of Nerja cave, portable figurative art was found in the archaeological deposits in the entrance rooms^45^.

Finally, phases 10–12 (Early Neolithic, Recent Neolithic and Copper Age) could be related to the burial activities documented for these periods. These activities include the deposition of human skeletal remains, ceramics and/or personal ornaments^11,46^. In this paper, we provide new chronological data on these incursions, which run from the Early Neolithic to the Copper Age, as well as information on their topographical distribution, preferably linked to the Lower Galleries, although two specific explorations of the upper parts of the cave were also identified (samples 12, 19). In other words, the visits concerning Recent Prehistory are not restricted to burial activities in the Lower Galleries, but are also due to exploratory actions, not to mention the execution of cave art linked to these dates and present in one of the external rooms (specifically, the schematic anthropomorphs in the Torca room). Furthermore, the confirmation, through the analysis of the micro-levels of soot present in the stalagmite, of at least 64 visits constitutes a real novelty for the understanding of the use of the subterranean environment during Recent Prehistory. In particular, between the Early Neolithic and the Recent Neolithic (phases 10 and 11) there was an average of one visit inside the cave every 35 years (considering average chronological values of 95.4% confidence intervals).

Dates 67 and 68, relating to an antiquity greater than 40,000 years old, are controversial dates, which deserve to be analysed in depth, as they imply significant interpretative changes for the Prehistory of the southern Iberian Peninsula. These samples were found at two different points in the Upper Galleries (in the Pisciform and Hercules rooms), in sectors located more than 1 km from the entrance and extremely difficult to access. Anthropic occupation of the subterranean environment existed since at least the Middle Pleistocene (ca. 176,000 years BP), as attested by evidence located more than 300 m from the entrance in the Bruniquel cave (Aveyron, France). Evidence includes the use of lighting systems, necessary for subterranean appropriation, by Neanderthal groups, the geomorphological modification of space and the construction of structures using speleothems^1^. The evidence from Nerja would be a significant novelty, as it would certify the ability of another ancient human species (*Homo neanderthalensis*), not only to frequent areas in total darkness and far from the entrance (as in the case of the Bruniquel cave), but also to overcome extremely difficult speleological obstacles inside caves.

These dates were among the first we made and were not subjected to anthracological analysis prior to dating, i.e. they were not subjected to microscopic study to confirm their anthropic origin. However, we do have δ^13^C analysis of both samples (Supplementary information S2), one of which (dating 68), falls within the range for carbonised organic matter (− 29 to 21‰). As this parameter is generally used to assess fractionation during ^14^C measurement, it is difficult, without an independent analysis apart from dating, to reach a conclusion. For all these reasons^47–49^, we consider this result insufficient to accept (for the moment) these revolutionary dates; we also consider it essential to undertake other interdisciplinary analyses to definitively confirm or reject the existence of Middle Palaeolithic visits inside Nerja cave (Supplementary information S3). We therefore began the chronometric analysis of the carbonates associated with the parietal art of Nerja^17,22,41,44^, which has offered novel methodological aspects on U/Th and ^14^C dating, despite no conclusive data on the Neanderthal chronology of any of the graphic motifs located inside the Nerja cave.

On the other hand, we do not rule out the possibility that in the future the number of phases of visits will be extended, for example with phases of occupation related to the last hunter-gatherers (Epipalaeolithic phases), already recorded in the stratigraphic sequence of the entrance rooms and common in the internal areas of other caves decorated with Palaeolithic art (some of them in areas very far from the entrance and/or with very complicated access)^50–54^. Nevertheless, and in the same way as for the previous case referring to the beginning of the visits from the Middle Palaeolithic, and although we have two dates that could testify to Epipalaeolithic visits (n. 20, 21), for the moment, we prefer not to take this information into account, as it did not pass the validity test to which we subjected all the dates before the Bayesian study (for more information, see Supplementary information S1, S2 and S3). It should also be remembered that our samples come from a particular context with no stratigraphic data to support the dates. Furthermore, the application of the analysis of soot levels on carbonate deposits of Palaeolithic chronology could extend the minimum number of visits. Previous studies confirmed the existence of carbonate formations for Palaeolithic chronology in Nerja Cave^41^, in particular thin calcium carbonate films on some paintings, so it would be possible to apply this state-of-the-art methodology to the Pleistocene occupation period as well.

In terms of the anthracological data obtained, “*Pinus* tp. *sylvestris-nigra*” is the most frequent taxonomic identification (if we exclude the general characterisation of “indeterminate charcoals”), not only in the charcoal samples dated from inside the cave, but also in all the samples examined in relation to the fuel used for lighting in Nerja cave^12,13^. As already shown in previous studies, there is a repeated and almost exclusive use of this type of wood for cave raids and for lighting, especially during the Upper Palaeolithic. The environmental conditioning factor does not seem to be the main reason for this choice, because other species were available in the surroundings of the caves^55^, and other reasons for this choice have been proposed, linked to functional purposes (due the benefits of these resinous woods for cave lighting) and even cultural ones^56,57^.

Furthermore, thanks to the ^14^C dating included in this work, following anthracological analysis, we have verified that the choice of *Pinus* tp. *sylvestris*-*nigra* wood for lighting-related activities in Nerja cave is not only a wood that is preferentially used compared to other types available in the environment (*Pinus* tp. *pinea*-*pinaster*, *Juniperus* sp.)^12,54^, but also that it is not exclusive to a specific Palaeolithic phase or cultural period, but is a multiphasic selection. Specifically, the choice of this woody resource for lighting was recorded in 8 of the 9 Palaeolithic occupation phases documented in this work, between phases 2–9 (Gravettian—Upper Magdalenian) inclusive. Likewise, the repeated use over time of Pinus *sylvestris* for lighting has been previously proposed for the Chauvet cave, and for two different periods of occupation referring to the Aurignacian and Gravettian^56,57^. The anthracological data from this study further reinforce this idea of a preferential and cross-cultural choice of *Pinus* tp. *sylvestris*-*nigra* for lighting activities inside caves during the Upper Palaeolithic.

## Conclusions

In this work we carried out an interdisciplinary and chronometric study of different combustion residues (charcoal and microlayers of soot) located in the Internal Archaeological Context of Nerja cave. The charcoals are linked to the lighting systems used by prehistoric groups to enter the deep areas of the cave and were found mainly on the surface in the interior of the cave, although we also included in this work two samples of the carbonaceous pigment of two Palaeolithic signs. The ^14^C dating of 53 of these charcoals, following anthracological analysis and after passing an ad hoc validation test and a subsequent Bayesian analysis, identified 12 phases of prehistoric visits to the interior of the cave between 41,218 and 3299 cal BP, and enabled us to define the periods between phases and the duration of each phase. This has allowed us to extend the general chronological interval of the site, pushing back the origin of human occupation in Nerja cave by more than 10,000 years, as well as to identify chrono-cultures hitherto unknown in the cave, such as the Aurignacian, Lower Magdalenian and Middle Magdalenian, some of which are very little known for the context of the southern Iberian Peninsula.

The interdisciplinary analysis of the microlayers of soot present in a stalagmite of Holocene chronology allowed us not only to robustly characterise this type of remains but also to carry out a high-precision zoom on the last three phases of visits identified through Bayesian analysis, proving that these three phases contain a minimum of 64 distinct incursions. Based on this analysis, we propose that between the Early Neolithic and the Recent Neolithic (phases 10 and 11) there could have been a visit to the interior of the cave every 35 years on average (without excluding another rate of occupation for this interval). This level of chronological precision for prehistoric archaeology is truly ground-breaking.

The prehistoric subterranean activities of Nerja cave are not only heterogeneous in terms of chronological diversity, but also in terms of spatial extension, showing moments of advance and regression in relation to the surface and depth throughout the different phases of prehistoric use and visits to the interior of the cave. The topographic analysis of the charcoal dated and the soot microlayers shows that not all the spaces in the cave were used in the same periods, with the reiteration of visits to the Cataclysm Room (Lower Galleries) or the Raspador Platform (where the stalagmite was collected with the recorded soot layers), and the more restricted occupation of the Upper and deeper Galleries of the cave being particularly noteworthy.

A representative set of radiocarbon dates such as those presented in this work, linked to the combustion residues located in the deep areas of cavities with Palaeolithic Art, can offer information on the chronology of the art, although not in a direct or conclusive manner. This type of chronological information is useful as a complement to other data (archaeological, geomorphological, etc.), especially when direct dating of rock art is difficult to carry out, as it is made of inorganic material or, as in the case of some of the black paleolithic graphics of the Upper Galleries of Nerja cave, because the carbonised pigment is deeply embedded in the wall and impossible to sample using current techniques. The chronological determination of Palaeolithic art through indirect approaches is a complex task and it is essential to combine different methods and study strategies^22,41^.

The data from the anthracological analysis, prior to the dating of the charcoals, point to a specific and transcultural use of *Pinus* tp. *sylvestris-nigra* for activities linked to the lighting of the cave, at least between the Gravettian and Upper Magdalenian periods, and not linked to environmental restrictions. It can therefore be concluded that the choice of this type of wood for incursions into caves is a transcultural and multiphasic choice, at least during the time when this type of tree was available in the cave environment, and probably linked to the benefits of this type of resinous wood for lighting activities.

Overall, this work has demonstrated the potential of interdisciplinary and chronometric analysis of the Internal Archaeological Context, and in particular of charcoals and soot micro-levels linked to different prehistoric lighting systems, for a comprehensive understanding of the “lifespan” of a prehistoric cave.

## Supplementary Information

Supplementary Information.

## Data Availability

All data generated or analysed during this study are included in this published article [and its supplementary information files]. However, should any information that any researcher wishes to consult be missing, it will be made available through the corresponding author on reasonable request.
