# Supplementary material for: 35,000 years of recurrent visits inside Nerja cave (Andalusia, Spain) based on charcoals and soot micro-layers analyses
Source: Sci Rep. 2023 Apr 11;13:5901. doi: 10.1038/s41598-023-32544-1 (PMC10090096; doi:10.1038/s41598-023-32544-1)
Supplement: Supplementary file 1 — Supplementary Information. [file 41598_2023_32544_MOESM1_ESM.pdf]

Supplementary information

S1: Methodology.

S2: Compilation of the 14C dating (uncal. BP) from Inner Archaeological Context of the Nerja cave.

S3: Detailed explanation of why some samples are not included in the Bayesian analysis.

S4: Plot used in Bayesian analysis (Oxcal 4.4.)

S5: Calibrated ages, modelled ages, start-finish dates of each phase, number of years per phase and per interval between phases.

S6: Carbonate dating results from GN16/08 stalagmite with soot micro-levels.

S7: Interdisciplinary analysis on soot microlayers from GN16/08 stalagmite.

S8: Image of the geographic situation of the cave, with the location of the sites mentioned in the article and a photograph of the archaeological context inside the cave

S9: References in the supplementary information.

S1: Methodology.

The methodology of this study comprised the following phases:

1. CHARCOAL ANALYSIS

1.1. Sampling: All charcoals selected for dating were sampled individually using sterilised collection tools and containers and aluminium folds to avoid contamination during sampling. Large fragments were divided in situ into two independent samples, one for anthracological analysis and the other for carbon dating. As anthracological analysis does not contaminate the sample (Vernet et al. 1979, Badal et al. 2003, Carrión et al. 2018), it avoids adding additional "stress" to the charcoal from dating. Most of the paleolithic charcoals found inside the cave at the surface are highly fragile, and fall apart upon trying to obtain the three planes of anatomical analysis during anthracological analysis. Part of the sample is therefore lost during this examination in most cases.

The sample pot selected for the charcoal was adapted as far as possible to the fragment, so as to avoid excessive movement of the sample inside the pot during transport. As already mentioned, these charcoals usually have a low cohesion and the slightest handling or transport causes them to crumble, which is a major drawback for anthracological and dendro-anthracological analysis.

1.2. Anthracological analysis: Anthracological analyses were conducted to determine the taxonomy of the wood charcoal. To identify the anatomical and biometric properties of the wood specimens, clean cuts were made on each fragment to obtain transversal, tangential and radial sections. The classification of each specimen and the alterations were determined by comparison with various reference sources and papers (Schweingruber 1990, Vernet et al. 2001, García et al. 2003). A light microscope was used with light reflected to a light-dark reflection field at magnifications ranging from 40 to 1000x. To carry out a more detailed observation of the sample and reach higher magnifications, we used a scanning electron microscope (SEM). Most of the observations were performed at the Central Service for Research Support (SCAI) at the University of Cordoba (UCO) using a model JEOL JSM 7800F, after gold-plating metallisation of the surface of the samples to obtain better quality images. The samples were placed in several cylindrical SEM sample carriers and were affixed to conductive carbon tape.

Anthracological identification prior to ^{14}C dating of wood charcoals does not alter the dating and is essential to know which woody species or genus is being dated, to identify the contamination of the sample with organic modern material, to detect charcoals linked to stratigraphic intrusions (in the case of stratigraphic sequences), as well as to use the date obtained to follow the history and presence of the plant in each region, and to determine which part of the wood was used (branches have less old-wood effect than the core of a timber) (Vernet et al. 1979, Badal et al. 2003, Carrión et al. 2018).

In the context of our study (Inner Archaeological Context of the cave), where most of the wood charcoals are found on the surface and have no stratigraphic relationship with other archaeological remains, the selection of taxa for dating that are presumably consistent at the paleoclimatic level with the assumed chronology of the art present in the cave will provide, with a high probability, a chronology linked to that cultural phase. The selection of taxa inconsistent with the dates of the art representations, on the other hand, will make it possible to determine different times of human presence in the cave, and even to determine the visualisation of the art by groups other than the artists.

In the endokarst there are numerous substances that may have a macroscopic appearance similar to that of charcoal (guano, decomposing organic matter, etc.). For this reason, micromorphological identification of the sample is essential to know what we are dating and its possible relation with the anthropic frequentation of the cave. Sampling by a specialist in Anthracology will considerably minimise errors in this respect. In addition, taphonomic analysis of the charcoal can alert us to possible post-depositional fungal contamination of the charcoal sample, for example, related to endokarst microbiota. In this situation, it is advisable to choose a different sample for dating or to instruct the laboratory to intensify the pre-treatment of the sample. If it cannot withstand an aggressive humic acid pre-treatment (depending on the consistency and size of each sample), it is recommended to take the result in *ante quem* terms.

1.3. Chemical pre-treatments and ^{14}C-AMS dating: ^{14}C-AMS dating was carried out in two different laboratories: LSCE (Laboratory for Sciences of Climate and Environment, CEA-CNRS-UVSQ, France), and Beta Analytic (USA). The ABA pre-treatment protocol was applied in the two labs. Specifically, in the LSCE laboratory before the graphitised sample is measured with a mass spectrometer, it is observed through a binocular magnifier to eliminate any visible foreign element. Next, a chemical treatment is applied to remove any contamination with foreign carbons that could lead to erroneous results. The mechanism is the following: a series of treatments with hydrochloric acid (0.5N) to eliminate carbonates, along with a basic treatment with sodium hydroxide (0.1N) to eliminate humic and fulvic acids (in other words, the bacteria and plant material from the soil). Hydrochloric acid is also used to eliminate the carbon gas absorbed during the treatment with the base. This was all carried out at approximately 80ºC. However, depending on the consistency of the sample, its condition, and the behaviour with the reagents, the treatment may be more or less aggressive (Valladas et al. 1999). The sample is then transformed to graphite (Cottereau et al. 2007) before analysis by AMS (LMC14 - Artemis National French facility).

In the Beta Analytic Laboratory, "*before radiocarbon dating, the sample is first gently crushed then dispersed in deionized water. It is then washed with hot HCl acid to eliminate carbonates followed by an alkali wash (NaOH) to remove secondary organic acids. The alkali wash is followed by a final acid rinse to neutralize the solution before drying. Chemical concentrations, temperatures, exposure times, and number of repetitions depend on the sample submitted. Each chemical solution is neutralized prior to application of the next. During these serial rinses, mechanical contaminants such as associated sediments and rootlets are eliminated*" (https://www.radiocarbon.com, consulted 23/02/20). For more information on the conversion of sample Carbon to

Graphite and other methodological aspects followed by this laboratory, please consult the following link: https://radiocarbon-1bfd8.kxcdn.com/PDF/AMS-Methodology.pdf

1.4. Bayesian analysis and prerequisites.

1.4.1. Validity test: the conditions that a radiocarbon date should fulfil to be considered valid for Bayesian analysis are the following:

- *Analytical prerequisite*: It is assumed that the dating procedure undertaken by the laboratory was correctly performed (as regards pre-treatment, measurement of radiocarbon content, etc.), and that the result was validated. Due to the existence of consecutive dates, and with the intention of obtaining a chronology as precise as possible for each phase, we set 1.8% as the maximum standard deviation. We considered it more appropriate to express the maximum error as a percentage rather than as a fixed digit, since we are working with an extensive chronological range, covering the Pleistocene and the Holocene.

- *Physicochemical prerequisite*: the ability of the material to be dated to produce an appropriate radiocarbon value to achieve our objective, i.e. the sample must be of organic origin and related to residues of prehistoric occupations within the cave. A microscopic examination (anthracological analysis for charcoal) was therefore carried out before dating, in order to know the nature of the samples to be dated and determine whether they do indeed correspond to prehistoric residues. The dating carried out by Beta Analytic also uses $\delta\ ^{13}C$ analysis to approximate the nature of the samples (by IRMS). However, given the lack of consensus on the ability of this analysis to provide definitive data in this regard (Jouve 2013, Fontugne et al. 2014, Audiard et al. 2018), we did not consider these results to be conclusive, although they should be considered together with the results of other analyses.

1.4.2. High precision Bayesian analysis: to model the radiocarbon results included in this work, we used the online software ©Oxcal 4.4 to accomplish two tasks: calibrating the radiocarbon dates in calendar years using the IntCal20 calibration curve (Reimer et al. 2020), thus making all the chronological information handled homogeneous; identifying and constraining the different phases of internal human occupation in the cave, using Bayesian statistics. This determination facilitated a chronological comparison (developed in the discussion section) between the anthropization of the different spaces in the cave (access chambers and interior chambers), as well as a chronology for the Palaeolithic art (based on the techno-stylistic information and the direct and indirect dating available), to obtain dates related to prehistoric human presence inside the cave.

The Bayesian analysis integrates the archaeological information ("prior") as *a priori* to the physical measurement (a priori hypothesis = standardised probability + ensuing hypothesis). The relative time ("prior") expressed in archaeological data is related to the independent physical measurement (dating) through the law of probability, modelling *a posteriori* the temporal distribution gathering of all available chronological information on the analysed sample. If the index of agreement between the model and the chronological (a priori) data is greater than or equal to 60%, this implies that the model could be true and that there is no basis for rejecting it (Bronk Ramsey 2009a, Quiles et al. 2014).

The different phases were defined according to the presence of archaeological materials (and not through the radiocarbon dating results) of each of the proposed phases in the stratigraphic deposits of the entrance rooms of the cave, or, failing that, to the presence of this type of cultural material in the regional context. For this purpose, using OxCal4.4, we sequenced these suggested Phases, limited by Boundaries determining the temporal distributions associated with the changes of phase. We also used the Outlier Charcoal model (Bronk Ramsey 2009b) to properly down-weight outlier samples, taking into account the long life of this material. This allowed us first to confirm the plausibility of the model, then to determine the start date (beginning of the boundary) and the end date (end of the boundary) for the different phases of human presence within the cave. The duration of each phase was calculated with the Span and Interval command. Span calculates the duration between the earliest and latest dating and Interval between the boundary start and boundary end of each phase. We also used the Interval command to calculate the transition period between phases (Bronk Ramsey, 1995, 2001; Bronk Ramsey and Lee 2013, Morrell 2019). Finally, the humic fraction dating was included in the model using the *Before* formula (see *Supplementary information S3* for the plot used*)*.

2. SOOT MICRO-LAYER STUDY:

2.1. Fuliginochronological analysis.

The use of fire for lighting is necessary for deep cave exploration (Medina-Alcaide et al. 2021), and lighting systems are responsible for soot production. Speleothems are archives that form in caves and are likely to record this soot (see Vandevelde et al. 2018 and references therein for various examples from around the world); their fuliginochronological analysis allows human visits to the site to be recorded in their chronological order. These chronologies can be materialised in the form of barcodes, where soot films are represented as solid lines, and uncertain (hardly observable) soot films or microcharcoal alignments are indicated as dotted lines (Figures 1; S6-7) in order to determine the minimum number of occupations over a given period, and to identify occupation phases (separated by stops in the use of the site or characterised by changes in the rhythm of occupation – Vandevelde et al. 2018, Vandevelde 2021). These carbonate archives are also directly datable (see Vandevelde et al. 2020 and references therein), as shown by numerous studies and in particular the recent work carried out at Nerja cave (Pons-Branchu et al. 2022), enabling these occupation chronicles to be placed in calendar time.

It turns out that stalagmite GN16/08 in the Nerja cave records soot deposits. The identification of the black deposits as soot was confirmed by Raman spectroscopy (Figure 3, S6) and TEM analysis (Figures 4-5, S6), see below. The fuliginochronological analysis (presented below) made it possible to reconstruct the chronicle of occupations over two phases, which were directly dated by radiocarbon (cf. Supplementary information S4).

For the fuliginochronological analysis of this stalagmite, a polished section and the corresponding thin section made in the growth axis of the sample were observed under optical microscopy. The microscopy analysis and images were done with the equipment of the ArchéoScopie Platform of the MSH Mondes (USR 3225 CNRS, Nanterre, France).

Raw data collection was performed using the LnSeq module of the Datawald platform (GL Conception; details in Vandevelde et al. 2017).

2.2. Soot characterization:

2.2.1. Raman micro-spectroscopy analysis: The identification of black layers as carbonaceous matter was confirmed unequivocally by Raman micro-spectroscopy performed on the central soot bundle of an unpolished section of GN16/08 at the Laboratoire de Géologie de l'ENS (École Normale supérieure, Paris, France). A Renishaw® inVia Raman microspectrometer was used with a Cobolt 514 nm laser. Data acquisition was performed with 5 % laser intensity (2.5 mW) and a 20 s time exposure. Figure 3, S6 shows two broad peaks around 1350 and 1600 cm-1 that correspond to D (defect) and G (graphite) bands of polycyclic aromatic materials (Sadezky et al. 2005, Deldicque et al. 2016). These are typical signals of solid products of the carbonization process. The spectrum shape is typical of soot. The third peak recorded at 1085 cm-1 is characteristic of calcite (Martínez-Ramírez et al. 2003, Bonneau et al. 2012). The concomitant presence of these three peaks shows that the polycyclic aromatic material is well cemented in the carbonate matrix that it stains (Vandevelde et al. 2017).

2.2.2. Transmission electron microscopy-Energy Dispersive X-ray spectroscopy (TEM-EDX) analysis: TEM-EDX allows the characterization of the anatomical structure of solid samples at nanometric scale and the production of analytical maps. This tool was previously used to characterize soot layers of speleothems from the Dominica cave in Slovakia (Pawlyta and Hercman 2016). Our sample, a powder extracted by scraping from one black level of the stalagmite with a scalpel, was prepared following Pawlyta and Hercman (2016) from characterization of the soot micro-level inside the GN16/08 stalagmite: after acid dissolution with 1M HCl, the sample was rinsed with distilled water, and finally filtrated to obtain nano-sized particles to be analyzed using TEM-EDX (Figures 4-5, S6). TEM-EDX analyses were performed at the University of Malaga, using a FEI Talos F200X equipment. The TEM images show spherical nanoparticles with an onion-like (concentric) nanostructure confirming that the carbonaceous material identified by Raman spectroscopy is soot.

2.3. Stalagmite radiocarbon dating: The $CaCO_3$ samples from the stalagmite were hydrolyzed with H_3PO_4 to obtain CO_2 and then converted to graphite as described by Tisnerat et al. (2001) and Dumoulin et al. (2017). They were measured at the Artemis AMS-French National facility (CEA Saclay, LMC14; Moreau et al. 2020). The ^{14}C measurements were corrected for isotopic fractionation according to the $\delta^{13}C$ values measured on the AMS, following international recommendations (Mook and Van Der Plicht 1999).

Previous studies at Nerja cave showed that the dead carbon proportion is low, in most cases close to 0 % and always between 0 to 10% for $CaCO_3$ samples from several sectors of the cave (Sanchidrián et al. 2017, Valladas et al. 2017, Pons-Branchu et al. 2022). Radiocarbon data were corrected for 10% of dead carbon and then calibrated using OxCal 4.4 (Bronk Ramsey 2006) and the Intcal20 data (Reimer et al. 2020) assuming 0 or 10% of dead carbon.

S2: Compilation of the 14C-AMS dating (uncal. BP) from Inner Archaeological Context of the Nerja cave.

Nº	LABORATORY REF.	GALLERY	CHAMBER	NATURE	δ13C (o/oo)	BP.	CULTURE	REFERENCE
**1.**	GifA13395/SacA36457	Upper	Cocina-Pisc.	not analysed		Modern	Modern / historic	Unpublished
**2.**	GifA13137/SacA33776	Upper	Cocina-Pisc.	not analysed		Modern	Modern / historic	Unpublished
**3.**	GifA13140/SacA33767	Upper	Cocina-Pisc.	not analysed		Modern	Modern / historic	Unpublished
**4.**	GifA13142/SacA33760	Upper	Cocina-Pisc.	not analysed		Modern	Modern / historic	Unpublished
**5.**	Beta-382316	Upper	Cataclismo	not analysed	-13.3	Modern	Modern / historic	Unpublished
**6.**	Beta-438093	Upper	Hércules	not analysed	-25.2	123 ± 50	Modern / historic	Unpublished
**7.**	Beta-444197	Upper	Lanza	not analysed	-25.6	160 ± 30	Modern / historic	Unpublished
**8.**	GifA13135/SacA33763	Upper	Cocina-Pisc.	not analysed		280 ± 35	Modern / historic	Unpublished
**9.**	GifA13141/SacA33768	Upper	Cocina-Pisc.	not analysed		500 ± 80	Modern / historic	Unpublished
**10.**	GifA13138/SacA33765	Upper	Cocina-Pisc.	not analysed		1640 ± 60	Modern / historic	Unpublished
**11.**	GifA13397/SacA36458	Lower	Cascada	*Pinus* sp.		3590 ± 35	Chalcolithic	Medina et al. 2015
**12.**	Beta-396387	Upper	Hércules	*Pinus* cf. tp. *pinea-pinaster*	-23.4	4410 ± 30	Chalcolithic	Unpublished
**13.**	Beta-277743	Lower	Cataclismo	Indeterminate charcoal	-29	4710 ± 40	Chalcolithic	Unpublished
**14.**	Beta-347462	Lower	Cascada	*Pinus* sp.	-25.7	5160 ± 30	Recent Neolithic	Medina et al. 2015
**15.**	GifA13408/SacA36468	Lower	Cataclismo	Indeterminate charcoal		5450 ± 30	Recent Neolithic	Unpublished
**16.**	Beta-270018	Lower	Cataclismo	Indeterminate charcoal	-23	5770 ± 40	Recent Neolithic	Aguilera et al. 2015
**17.**	Beta-270019	Lower	Cataclismo	Indeterminate charcoal	-24.4	6040 ± 40	Ancient Neolithic	Aguilera et al. 2015
**18.**	Beta-271213	Lower	Cataclismo	Indeterminate charcoal	-21.7	6230 ± 40	Ancient Neolithic	Aguilera et al. 2015
**19.**	Beta-396385	Upper	Ciervo	Conifer	-27.1	6730 ± 30	Ancient Neolithic	Unpublished
**20.**	Beta-382314	Upper	Ciervo	not analysed	-24.4	8530 ± 30	Epipaleolithic	Unpublished
**21.**	Beta-298422	Upper	Hércules	not analysed	-28.6	9660 ± 50	Epipaleolithic	Unpublished
**22.**	GifA15161/SacA43842	Lower	Cataclismo	*Pinus* tp. *sylvestris-nigra*		12880 ± 60	Upper Magdalenian	Unpublished
**23.**	Beta-298418	Lower	Cataclismo	Indeterminate charcoal	-24.3	12890 ± 60	Upper Magdalenian	Medina et al. 2015
**24.**	GifA13407/SacA36467	Lower	Cataclismo	Indeterminate charcoal		13120 ± 110	Middle Magdalenian	Unpublished
**25.**	GifA15926/SacA44820	Lower	Cataclismo	*Pinus* tp. *sylvestris-nigra*		13270 ± 70	Middle Magdalenian	Unpublished
**26.**	Beta-342842	Lower	Cataclismo	*Pinus* cf. tp. *sylvestris-nigra*	-24.7	13380 ± 60	Middle Magdalenian	Medina et al. 2015
**27.**	Beta-342844	Lower	Cataclismo	Indeterminate charcoal	-24.6	13490 ± 50	Middle Magdalenian	Medina et al. 2015
**28.**	Beta-342843	Lower	Cataclismo	Indeterminate charcoal	-22.8	13920 ± 60	Middle Magdalenian	Medina et al. 2015
**29.**	GifA13139/SacA33766	Lower	Cataclismo	Indeterminate charcoal		14110 ± 60	Middle Magdalenian	Unpublished
**30.**	Beta-438092	Lower	Cataclismo	Indeterminate charcoal	-22.8	14320 ±60	Middle Magdalenian	Unpublished
**31.**	Beta-270020	Upper	Subida	Indeterminate charcoal		14320 ± 90	Middle Magdalenian	Medina et al. 2015
**32.**	Beta-396386	Upper	Cataclismo	Indeterminate charcoal	-24	15290 ± 50	Lower Magdalenian	Unpublished
33.	*GifA15158/SacA43839*	*Upper*	*Cocina-Pisc.*	Conifer		*15650 ± 300*	*Lower Magdalenian*	*Sanchidrián et al. 2017*

34.	*GifA13398/SacA36459*	*Upper*	*Cocina-Pisc.*	*Pinus tp. sylvestris-nigra*		*15790 ± 460*	*Lower Magdalenian*	*Sanchidrián et al. 2017*
**35.**	Beta-396388	Upper	Hércules	Indeterminate charcoal	-23.8	16160 ± 50	Lower Magdalenian	Unpublished
**36.**	Beta-438096	Upper	Subida	Indeterminate charcoal	-24.9	16270 ± 60	Lower Magdalenian	Unpublished
**37.**	GifA15165/SacA43843	Upper	Cocina-Pisc.	charcoal/humic fraction		16840 ±110	Upper Solutrean	Unpublished
**38.**	GifA15162/SacA43844	Upper	Cocina-Pisc.	charcoal/humic fraction		17310 ± 380	Upper Solutrean	Unpublished
**39.**	GifA15156/SacA43837	Upper	Cocina-Pisc.	*Pinus* sp.		17680 ± 100	Upper Solutrean	Unpublished
**40.**	Beta-396390	Upper	Hércules	*Pinus* tp. *sylvestris-nigra*	-23.8	17720 ± 70	Upper Solutrean	Unpublished
**41.**	Beta-298421	Upper	Hércules	*Pinus* tp. *sylvestris-nigra*	-24.3	17900 ± 90	Upper Solutrean	Unpublished
**42.**	Beta-438329	Upper	Inmensidad	*Pinus* tp. *sylvestris-nigra*	-24.1	18350 ± 70	Upper Solutrean	Unpublished
**43.**	Beta-438097	Upper	Ciervo	*Pinus* tp. *sylvestris-nigra*	-22.8	18540 ± 60	Upper Solutrean	Unpublished
**44.**	GifA15159/SacA43840	Upper	Cocina-Pisc.	charcoal/humic fraction		19440 ± 200	Middle Solutrean	Unpublished
**45.**	GifA438095	Upper	Subida	Indeterminate charcoal		19390 ± 70	Middle Solutrean	Unpublished
**46.**	Beta-396392	Upper	Cocina-Pisc.	Conifer	-23.4	19620 ± 70	Middle Solutrean	Unpublished
**47.**	Beta-438091	Upper	Inmensidad	Indeterminate charcoal	-23.8	19700 ± 80	Middle Solutrean	Unpublished
**48.**	GifA98191	Upper	Ciervo	Indeterminate charcoal		19900 ± 210	Middle Solutrean	Sanchidrián et al. 2001
**49.**	Beta-396389	Upper	Hércules	*Pinus* tp. *sylvestris-nigra*	-24.9	20050 ± 80	Middle Solutrean	Unpublished
**50.**	Beta-396394	Upper	Hércules	Conifer	-24.1	20430 ± 70	Middle Solutrean	Unpublished
**51.**	Beta-438090	Upper	Inmensidad	*Pinus* tp. *sylvestris-nigra*	-25.7	20720 ± 70	Middle Solutrean	Unpublished
**52.**	Beta-271212	Lower	Cataclismo	Indeterminate charcoal	-24	20980 ± 100	Middle Solutrean	Romero et al. 2012
**53.**	Beta-438094	Lower	Cataclismo	Indeterminate charcoal	-26.3	21370 ± 90	Lower Solutrean	Unpublished
**54.**	GifA15160/SacA43841	Upper	Cocina-Pisc.	Conifer		21580 ± 150	Lower Solutrean	Unpublished
**55.**	Beta-347457	Lower	Cataclismo	*Pinus* tp. *sylvestris-nigra*	-23.5	21900 ± 90	Lower Solutrean	Medina et al. 2015
**56.**	GifA15927/SacA44821	Lower	Cataclismo	*Pinus* tp. *sylvestris-nigra*		23350 ± 190	Gravettian	Unpublished
**57.**	Beta-271211	Lower	Cataclismo	*Pinus* tp. *sylvestris-nigra*	-23.9	23800 ± 140	Gravettian	Medina et al. 2015
**58.**	Beta-298419	Lower	Cataclismo	*Pinus* cf. tp. *sylvestris-nigra*	-24.8	23880 ± 130	Gravettian	Medina et al. 2015
**59.**	GifA15925SacA44819	Lower	Cataclismo	Indeterminate charcoal		24090 ± 160	Gravettian	Unpublished
**60.**	Beta-277744	Lower	Cataclismo	Indeterminate charcoal	-23.2	24130 ± 140	Gravettian	Medina et al. 2015
**61.**	GifA13410/SacA36470	Lower	Cataclismo	Indeterminate charcoal		24220 ± 180	Gravettian	Unpublished
**62.**	GifA13406/SacA36466	Lower	Cataclismo	Indeterminate charcoal		24370 ± 170	Gravettian	Unpublished
**63.**	GifA13409/SacA36469	Lower	Cataclismo	Indeterminate charcoal		24430 ± 180	Gravettian	Unpublished
**64.**				Indeterminate charcoal				
**65.**	Beta-306992	Lower	Cataclismo	*Pinus* cf. tp. *sylvestris-nigra*	-23.2	29650 ± 160	Aurignacian	Medina et al. 2015
**66.**	Beta-277745	Lower	Cataclismo	Indeterminate charcoal	-24	35320 ± 360	Aurignacian	Medina et al. 2015
**67.**	Beta-298423	Lower	Hércules	not analysed	-29.7	42490 ± 630	Transitional UP/MP	Unpublished
**68.**	Beta-298425	Lower	Cocina-Pisc.	not analysed	-23.1	>43500	Mousterian	Unpublished

Table 1, S2 – Compilation of the 14C-AMS dating (uncal. BP) from Inner Archaeological Context of the Nerja cave, with indication of the room of provenance within the cave, the nature of the remains, δ13C value, dating result (uncal. BP), chronoculture to which it is thought to which correspond in the regional context and bibliographical reference (if already published). In red the dating excluded in the Bayesian model due to lack of anthracological characterisation prior to dating (we are not sure if what has been dated corresponds to residues from a pre-historic visit to the interior of the cavity, or if it is another endokarst organic residue), in grey the datings of the humic fraction (but included in the Bayesian analysis with the "Before" formula), and in green the datings excluded because they have a precision range >1.8%%. Directly dated black marks in the wall are shown in italics.

S3: Detailed explanation of why some samples are not included in the Bayesian analysis.

Following our "validation test" (supplementary information S1.4), **samples 1-10** were excluded as being related to contemporary use of the cavity and outside our prehistoric study objective. In addition, they were dated before anthracological identification, so we are not sure if the sample corresponds to combustion residues or if it is another endokarstic organic residue.

Sample 20-21 were excluded because they were dated prior to anthracological identification of the sample, so we are unsure whether they are anthropogenic residues from a prehistoric internal visit or another endokarst organic residue. The δ13C analysis (Supplementary Information -S2) of the samples falls within the range corresponding to carbonised organic matter (-29-21‰). Dating by Beta Analytic also uses the δ13C analysis to approximate the nature of the samples (by IRMS). However, as this parameter is generally used to assess fractionation during 14C measurement, it is difficult, without independent analysis apart from dating, to reach a conclusion. For all these reasons, we consider this result insufficient to accept (for the moment) these unpublished dates for the internal frequentation of the cave.

Sample 64 has been discarded for having a low level of precision and a deviation range of more than 1.8%. In order to obtain the most precise chronology possible for each phase, we set this percentage as the maximum standard deviation. We consider it more appropriate to express the maximum error as a percentage than as a fixed digit, since we are working with an extensive chronological range, spanning the Pleistocene and Holocene.

Samples 67-68 were among the first samples we made and were not subjected to anthracological analysis prior to dating, i.e., they were not subjected to microscopic study to confirm their anthropogenic origin. However, we do have δ13C analyses of both samples (Supplementary Information -S7), one of which (dating 68), falls within the range corresponding to carbonised organic matter (-29-21 ‰). As we have indicated above for dates 20-21, we consider this result insufficient to definitively confirm or reject the origen antropics visits inside the Nerja cave (Supplementary Information -S3). For this reason, we began the chronometric analysis of the carbonates associated with the parietal art of Nerja (Sanchidrián et al. 2017, Valladas et al. 2017, Pons-Branchu et al. 2020, 2022,), which has offered novel methodological aspects on the U/Th and 14C dating, despite not having conclusive data on the Neanderthal chronology of any of the graphic motifs located in the interior of the Nerja cave. Furthermore, on the dating 68 the laboratory indicated the following: "*The 14C activity was extremely low and almost identical to the background signal. In these cases, the indeterminate errors associated with the background add unmeasurable uncertainty to the result. Always, the result must be considered along* ***with other lines of evidence****. The most conservative interpretation of the age is infinite*".

S4: Plot used in Bayesian analysis (Oxcal 4.4).

```
Plot()
 {
  Outlier_Model("Charcoal",Exp(1,-10,0),U(0,3),"t");
  Sequence()
  {
   Boundary("Start Phase 1")
   {
    color="green";
   };
   Phase("1")
   {
    R_Date("Beta-277745", 35320, 360)
    {
     Outlier("Charcoal",1);
    };
    Span("Span of dates F1");
    Interval("Duration F1");
   };
   Boundary("End Phase 1")
   {
    color="blue";
   };
   Interval("Transition Phase 1/2");
   Boundary("Start Phase 2")
   {
    color="blue";
   };
   Phase("2")
   {
    R_Date("Beta-306992", 29650, 160)
    {
     Outlier("Charcoal",1);
    };
    Span("Span of dates  F2");
    Interval("Duration F2");
   };
   Boundary("End Phase 2")
   {
    color="blue";
   };
   Interval("Transition Phase 2/3");
   Boundary("Start Phase 3")
   {
    color="blue";
   };
   Phase("3")
   {
    R_Date("GifA13409/SacA36469", 24430, 180)
    {
     Outlier("Charcoal",1);
    };
    R_Date("GifA13406/SacA36466", 24370, 170)
    {
     Outlier("Charcoal",1);
    };
    R_Date("GifA13410/SacA36470", 24220, 180)
    {
     Outlier("Charcoal",1);
    };
    R_Date("Beta-277744", 24130, 140)
    {
     Outlier("Charcoal",1);
    };
    R_Date("GifA15925SacA44819", 24090, 160)
    {
     Outlier("Charcoal",1);
    };
    R_Date("Beta-298419", 23881, 130)
    {
     Outlier("Charcoal",1);
    };
```

```
 R_Date("Beta-271211", 23800, 140)
 {
  Outlier("Charcoal",1);
 };
 R_Date("GifA15927/SacA44821", 23350, 190)
 {
  Outlier("Charcoal",1);
 };
 Span("Span of dates F3");
 Interval("Duration F3");
};
Boundary("End Phase 3")
{
 color="blue";
};
Interval("Transition Phase 3/4");
Boundary("Start Phase 4")
{
 color="blue";
};
Phase("4")
{
 R_Date("Beta-347457", 21900, 90)
 {
  Outlier("Charcoal",1);
 };
 R_Date("GifA15160/SacA43841", 21580, 150)
 {
  Outlier("Charcoal",1);
 };
 R_Date("Beta-438094", 21370, 90)
 {
  Outlier("Charcoal",1);
 };
 Span("Span of dates F4");
 Interval("Duration F4");
};
Boundary("End Phase 4")
{
 color="blue";
};
Interval("Transition Phase 4/5");
Boundary("Start Phase 5")
{
 color="blue";
};
Phase("5")
{
 R_Date("Beta-271212", 20980, 100)
 {
  Outlier("Charcoal",1);
 };
 R_Date("Beta-438090", 20720, 70)
 {
  Outlier("Charcoal",1);
 };
 R_Date("Beta-396394", 20430, 70)
 {
  Outlier("Charcoal",1);
 };
 R_Date("Beta-396389", 20050, 80)
 {
  Outlier("Charcoal",1);
 };
 R_Date("Beta-438091", 19700, 80)
 {
  Outlier("Charcoal",1);
 };
 R_Date("Beta-396392", 19620, 70)
 {
  Outlier("Charcoal",1);
 };
 R_Date("GifA438095", 19390, 70)
 {
```

```
  Outlier("Charcoal",1);
 };
 Before (R_Date("GifA15159", 19440 , 200))
 {
  Outlier("Charcoal",1);
 };
 Span("Span of dates F5");
 Interval("Duration F5");
};
Boundary("End Phase 5")
{
 color="blue";
};
Interval("Transition Phase 5/6");
Boundary("Start Phase 6")
{
 color="blue";
};
Phase("6")
{
 R_Date("Beta-438097", 18540, 70)
 {
  Outlier("Charcoal",1);
 };
 R_Date("Beta-438329", 18350, 70)
 {
  Outlier("Charcoal",1);
 };
 R_Date("Beta-298421", 17900, 90)
 {
  Outlier("Charcoal",1);
 };
 R_Date("Beta-396390", 17720, 70)
 {
  Outlier("Charcoal",1);
 };
 R_Date("GifA15156/SacA43837", 17680, 100)
 {
  Outlier("Charcoal",1);
 };
 Before (R_Date("GifA15162/SacA43844", 17310, 380))
 {
  Outlier("Charcoal",1);
 };
 Span("Span of dates F6");
 Interval("Duration F6");
};
Boundary("End Phase 6")
{
 color="blue";
};
Interval("Transition Phase 6/7");
Boundary("Start Phase 7")
{
 color="blue";
};
Phase("7")
{
 Before (R_Date("GifA15165/SacA43843", 16840, 110))
 {
  Outlier("Charcoal",1);
 };
 R_Date("Beta-438096", 16270, 60)
 {
  Outlier("Charcoal",1);
 };
 R_Date("Beta-396388", 16160, 50)
 {
  Outlier("Charcoal",1);
 };
 R_Date("GifA13398/SacA36459", 15790, 460)
 {
  Outlier("Charcoal",1);
 };
```

```
 R_Date("GifA15158/SacA43839", 15650, 300)
 {
  Outlier("Charcoal",1);
 };
 R_Date("Beta-396386", 15290, 50)
 {
  Outlier("Charcoal",1);
 };
 Span("Span of dates F7");
 Interval("Duration F7");
};
Boundary("End Phase 7")
{
 color="blue";
};
Interval("Transition Phase 7/8");
Boundary("Start Phase 8")
{
 color="blue";
};
Phase("8")
{
 R_Date("Beta-270020", 14320, 90)
 {
  Outlier("Charcoal",1);
 };
 R_Date("Beta-438092", 14320, 60)
 {
  Outlier("Charcoal",1);
 };
 R_Date("GifA13139/SacA33766", 14110, 60)
 {
  Outlier("Charcoal",1);
 };
 R_Date("Beta-342843", 13920, 60)
 {
  Outlier("Charcoal",1);
 };
 R_Date("Beta-342844", 13490, 50)
 {
  Outlier("Charcoal",1);
 };
 R_Date("Beta - 342842", 13380, 60)
 {
  Outlier("Charcoal",1);
 };
 R_Date("GifA15926/SacA44820", 13270, 70)
 {
  Outlier("Charcoal",1);
 };
 R_Date("GifA13407/SacA36467", 13120, 110)
 {
  Outlier("Charcoal",1);
 };
 Span("Span of dates F8");
 Interval("Duration F8");
};
Boundary("End Phase 8")
{
 color="blue";
};
Interval("Transition Phase 8/9");
Boundary("Start Phase 9")
{
 color="blue";
};
Phase("9")
{
 R_Date("Beta-298418", 12890, 60)
 {
  Outlier("Charcoal",1);
 };
 R_Date("GifA15161/SacA43842", 12880, 60)
 {
```

```
  Outlier("Charcoal",1);
 };
 Span("Span of dates F9");
 Interval("Duration F9");
};
Boundary("End Phase 9")
{
 color="blue";
};
Interval("Transition Phase 9/10");
Boundary("Start Phase 10")
{
 color="blue";
};
Phase("10")
{
 R_Date("Beta-396385", 6730, 30)
 {
  Outlier("Charcoal",1);
 };
 R_Date("Beta-271213", 6230, 40)
 {
  Outlier("Charcoal",1);
 };
 R_Date("Beta-270019", 6040, 40)
 {
  Outlier("Charcoal",1);
 };
 Span("Span of dates F10");
 Interval("Duration F10");
};
Boundary("End Phase 10")
{
 color="blue";
};
Interval("Transition Phase 10/11");
Boundary("Start Phase 11")
{
 color="blue";
};
Phase("11")
{
 R_Date("Beta-270018", 5770, 40)
 {
  Outlier("Charcoal",1);
 };
 R_Date("GifA13408/SacA36468", 5450, 30)
 {
  Outlier("Charcoal",1);
 };
 R_Date("Beta-347462", 5160, 30)
 {
  Outlier("Charcoal",1);
 };
 Span("Span of dates F11");
 Interval("Duration F11 ");
};
Boundary("End Phase 11")
{
 color="blue";
};
Interval("Transition Phase 11/12");
Boundary("Start Phase 12")
{
 color="blue";
};
Phase("12")
{
 R_Date("Beta-277743", 4710, 40)
 {
  Outlier("Charcoal",1);
 };
 R_Date("Beta-396387", 4410, 30)
 {
```

```
    Outlier("Charcoal",1);
   };
   R_Date("GifA13397/SacA36458", 3590, 35)
   {
    Outlier("Charcoal",1);
   };
   Span("Span of dates F12");
   Interval("Duration F12");
  };
  Boundary("End Phase 12")
  {
   color="green";
  };
 };
};
```

S5: Calibrated ages (INTCAL20 cal. BP), modelled ages (Oxcal 4.4.), start-finish dates of each phase, number of years per phase and per interval between phases.

SAMPLE<br>**BOUNDARY START PHASE**<br>**BOUNDARY END PHASE**<br>*INTERVAL DURATION PHASE (YEARS)*<br>**INTERVAL TRANSITION (YEARS)**	**UNMODELLED (CAL. BP, INTCAL20)**				**MODELLED (CAL. BP, INTCAL20)**				**Indices**<br>**Amodel=100**<br>**Aoverall=98**				
	**from**	**to**	**%**	**µ**	**from**	**to**	**%**	**µ**	**Acomb**	**A**	**L**	**P**	**C**
Outlier_Model Charcoal					*-558*	*3*	*95.4*	*-122*					*90.5*
Exp(1,-10,0)	*-3.19*	*-0.05*	*95.4*	*-105.042*				*-105.104*					*100*
U(0,3)	*2,21E-12*	*3*	*95.4*	*1.515*	*3,60E-12*	*2.595*	*95.4*	*166.195*		*100*			*38.6*
Sequence													
**Boundary Start Phase 1**					**44385**	**39318**	**95.4**	**41218**					94.9
Phase 1													
R_Date Beta-277745	41142	39729	95.4	40452	41101	39361	95.4	40231		97.7			97.1
Span of dates F1					*0*	*5*	*95.4*	*3*					100
Interval Duration F1					*0*	*6538*	*95.4*	*2401*					97.9
**Boundary End Phase 1**					**40906**	**35648**	**95.4**	**38818**					98
**Interval Transition Phase 1/2**					**0**	**5794**	**95.4**	**3016**					99.3
**Boundary Start Phase 2**					**39138**	**33742**	**95.4**	**35802**					98.3
Phase 2													
R_Date Beta-306992	34497	33867	95.4	34202	34497	33554	95.4	34077		99.9			95.2
Span of dates F2					*0*	*5*	*95.4*	*3*					100
Interval Duration F2					*0*	*8112*	*95.4*	*3637*					98.3
**Boundary End Phase 2**					**34325**	**29059**	**95.4**	**32165**					98.2
**Interval Transition Phase 2/3**					**223**	**5781**	**95.4**	**3539**					99
**Boundary Start Phase 3**					**29310**	**27889**	**95.4**	**28626**					91.6
Phase 3													
R_Date GifA13409/SacA36469	29122	28139	95.4	28649	28861	27691	95.4	28320		86.3			91.3
R_Date GifA13406/SacA36466	29036	28042	95.4	28567	28805	27690	95.4	28289		91.5			91.3
R_Date GifA13410/SacA36470	28793	27877	95.4	28379	28704	27658	95.4	28185		100.1			93
R_Date Beta-277744	28673	27885	95.4	28291	28627	27652	95.4	28136		101.2			93.2

R_Date GifA15925SacA44819	28660	27845	95.4	28254	28617	27630	95.4	28107		102.5			93.7
R_Date Beta-298419	28432	27743	95.4	28042	28416	27531	95.4	27945		100.9			95.5
R_Date Beta-271211	28353	27695	95.4	27971	28340	27474	95.4	27887		99.3			96.1
R_Date GifA15927/SacA44821	27813	27276	95.4	27549	28019	27211	95.4	27627		83.6			97.6
Span of dates F3					*214*	*1427*	*95.4*	*857*					97.8
Interval Duration F3					*130*	*2169*	*95.4*	*1181*					97.4
**Boundary End Phase 3**					**27935**	**26811**	**95.4**	**27445**					98
**Interval Transition Phase 3/4**					**288**	**1999**	**95.4**	**1258**					98.7
**Boundary Start Phase 4**					**27019**	**25585**	**95.4**	**26188**					87.7
Phase 4													
R_Date Beta-347457	26370	25930	95.4	26148	26321	25541	95.4	25936		98.4			85.2
R_Date GifA15160/SacA43841	26259	25641	95.4	25880	26072	25428	95.4	25794		107.9			89.6
R_Date Beta-438094	25934	25366	95.4	25737	25928	25454	95.4	25714		101.2			92.5
Span of dates F4					*0*	*583*	*95.4*	*267*					95.8
Interval Duration F4					*0*	*1519*	*95.4*	*628*					94.7
**Boundary End Phase 4**					**25881**	**25145**	**95.4**	**25560**					93.1
**Interval Transition Phase 4/5**					**0**	**818**	**95.4**	**334**					98
**Boundary Start Phase 5**					**25740**	**24602**	**95.4**	**25226**					91
Phase 5													
R_Date Beta-271212	25636	25078	95.4	25339	25554	24229	95.4	25016		101.3			88
R_Date Beta-438090	25195	24712	95.4	24987	25209	24183	95.4	24820		98.9			89.9
R_Date Beta-396394	24854	24252	95.4	24540	24886	23891	95.4	24413		100.2			94.6
R_Date Beta-396389	24250	23850	95.4	24051	24265	23492	95.4	23939		100			93.9
R_Date Beta-438091	23875	23382	95.4	23665	23866	23191	95.4	23584		103.4			93.2
R_Date Beta-396392	23822	23356	95.4	23575	23813	23167	95.4	23505		99.9			94.5
R_Date GifA438095	23742	23078	95.4	23401	23735	23027	95.4	23410		98.9			95.3
Before	...	-21823.2	95.4										
R_Date GifA15159	23809	23001	95.4	23424	23824	22700	95.4	23302		100.1			96.7
Span of dates F5					*1114*	*2291*	*95.4*	*1719*					96.4
Interval Duration F5					*1366*	*2944*	*95.4*	*2129*					97.9
**Boundary End Phase 5**					**23620**	**22521**	**95.4**	**23097**					97.4
**Interval Transition Phase 5/6**					**0**	**1226**	**95.4**	**609**					98.2
**Boundary Start Phase 6**					**23150**	**21633**	**95.4**	**22488**					91.3
Phase 6													

R_Date Beta-438097	22632	22301	95.4	22449	22575	21519	95.4	22232		103.4			87
R_Date Beta-438329	22435	22136	95.4	22291	22436	21532	95.4	22126		99.7			88
R_Date Beta-298421	22027	21427	95.4	21743	22033	21248	95.4	21655		101.6			95.1
R_Date Beta-396390	21821	21164	95.4	21514	21816	21093	95.4	21482		101.9			96.7
R_Date GifA15156/SacA43837	21809	21016	95.4	21418	21843	21004	95.4	21446		97.3			97.5
Before	...	-19956.8	95.4										
R_Date GifA15162/SacA43844	22055	20055	95.4	21011	22000	19848	95.4	20883		100.3			98.1
Span of dates F6					*253*	*1434*	*95.4*	*911*					96
Interval Duration F6					*17*	*2345*	*95.4*	*1357*					97
**Boundary End Phase 6**					**21660**	**20500**	**95.4**	**21131**					98.3
**Interval Transition Phase 6/7**					**0**	**1433**	**95.4**	**697**					97.9
**Boundary Start Phase 7**					**21223**	**19596**	**95.4**	**20435**					92.4
Phase 7													
Before	...	-18608.8	95.4										
R_Date GifA15165/SacA43843	20608	20022	95.4	20346	20557	19319	95.4	20095		94.1			89.1
R_Date Beta-438096	19846	19500	95.4	19660	19863	19054	95.4	19533		100.2			92.1
R_Date Beta-396388	19614	19359	95.4	19503	19614	18880	95.4	19381		100			89.4
R_Date GifA13398/SacA36459	20280	18231	95.4	19198	20108	18205	95.4	19113		105.1			98.3
R_Date GifA15158/SacA43839	19585	18274	95.4	18986	19582	18197	95.4	18912		102.8			97.6
R_Date Beta-396386	18754	18302	95.4	18522	18758	18150	95.4	18489		100.3			93.2
Span of dates F7					*711*	*1703*	*95.4*	*1168*					99.2
Interval Duration F7					*1318*	*3298*	*95.4*	*2284*					97.7
**Boundary End Phase 7**					**18678**	**17545**	**95.4**	**18151**					98.1
**Interval Transition Phase 7/8**					**0**	**1195**	**95.4**	**614**					99.5
**Boundary Start Phase 8**					**18161**	**16980**	**95.4**	**17537**					93.4
Phase 8													
R_Date Beta-270020	17801	17116	95.4	17461	17752	16628	95.4	17225		105			90.6
R_Date Beta-438092	17789	17148	95.4	17445	17717	16625	95.4	17234		107.1			89
R_Date GifA13139/SacA33766	17355	17030	95.4	17185	17373	16544	95.4	17049		100.4			92.2
R_Date Beta-342843	17075	16674	95.4	16906	17078	16294	95.4	16785		99.9			93
R_Date Beta-342844	16440	16062	95.4	16260	16442	15779	95.4	16160		100.3			93.7
R_Date Beta-342842	16304	15893	95.4	16104	16292	15691	95.4	16018		101			94.4
R_Date GifA15926/SacA44820	16180	15724	95.4	15945	16175	15595	95.4	15890		101.1			95
R_Date GifA13407/SacA36467	16062	15359	95.4	15735	16125	15487	95.4	15803		91.5			95.9

Span Span of dates F8					*1140*	*2013*	*95.4*	*1571*					98.2
Interval Duration F8					*1307*	*2562*	*95.4*	*1896*					99
**Boundary End Phase 8**					**15960**	**15309**	**95.4**	**15641**					94.6
**Interval Transition Phase 8/9**					**0**	**517**	**95.4**	**216**					99.9
**Boundary Start Phase 9**					**15788**	**15061**	**95.4**	**15424**					92.9
Phase 9													
R_Date Beta-298418	15614	15219	95.4	15413	15575	14870	95.4	15272		104.6			89.5
R_Date GifA15161/SacA43842	15604	15205	95.4	15401	15571	14866	95.4	15267		104.9			89.5
Span of dates F9					*0*	*329*	*95.4*	*96*					99.2
Interval Duration F9					*0*	*2826*	*95.4*	*690*					98.3
**Boundary End Phase 9**					**15549**	**12627**	**95.4**	**14734**					89.5
**Interval Transition Phase 9/10**					**3576**	**8422**	**95.4**	**6732**					96.1
**Boundary Start Phase 10**					**9943**	**6727**	**95.4**	**8003**					90.1
Phase 10													
R_Date Beta-396385	7665	7516	95.4	7595	7664	6831	95.4	7405		100			89.7
R_Date Beta-271213	7255	7003	95.4	7129	7253	6734	95.4	7031		99.5			90.3
R_Date Beta-270019	6994	6750	95.4	6885	7149	6569	95.4	6844		96.6			91.9
Span of dates F10					*5*	*861*	*95.4*	*581*					93.1
Interval Duration F10					*3*	*3284*	*95.4*	*1311*					95.7
**Boundary End Phase 10**					**6983**	**6249**	**95.4**	**6692**					92.8
**Interval Transition Phase 10/11**					**0**	**660**	**95.4**	**234**					97.1
**Boundary Start Phase 11**					**6831**	**5887**	**95.4**	**6458**					82.9
Phase 11													
R_Date Beta-270018	6667	6452	95.4	6569	6652	5797	95.4	6324		96.9			78.5
R_Date GifA13408/SacA36468	6300	6199	95.4	6247	6301	5779	95.4	6125		99			87.7
R_Date Beta-347462	5994	5765	95.4	5921	5993	5662	95.4	5874		98.9			89.7
Span of dates F11					*0*	*743*	*95.4*	*477*					83.6
Interval Duration F11					*11*	*1193*	*95.4*	*729*					92.6
**Boundary End Phase 11**					**5975**	**5395**	**95.4**	**5730**					96.4
**Interval Transition Phase 11/12**					**0**	**686**	**95.4**	**275**					98
**Boundary Start Phase 12**					**5875**	**4949**	**95.4**	**5455**					91.8
Phase 12													
R_Date Beta-277743	5578	5321	95.4	5432	5576	4639	95.4	5234		104.4			86.7
R_Date Beta-396387	5264	4865	95.4	4994	5264	4424	95.4	4869		100.3			91.1

R_Date GifA13397/SacA36458	4060	3729	95.4	3895	4065	3500	95.4	3818		99.1			93.8
Span of dates F12					*889*	*1818*	*95.4*	*1430*					91
Interval Duration F12					*1151*	*3576*	*95.4*	*2156*					97.6
**Boundary End Phase 12**					**3968**	**1993**	**95.4**	**3299**					98.4

S6: Carbonate dating results from stalagmite with soot micro-levels.

The chronology of the soot layers found in the GN16/08 stalagmite were determined by the analysis of $CaCO_3$ layers deposited before and after them. The soot layer at the base of the stalagmite was deposited between 7430 and 6740 cal BP (0% DCP); the soot layer from the upper level of the stalagmite was deposited between and 6840 and 3000 cal years BP assuming 0% of DCP year cal BP (Table 1, Figure 1, S5)

N° SacA	Depth mm from top	⍰^{13} C	pMC	Years Cal BP 0% DCP	Years Cal BP 10% DCP
SacA 57098	0.5	-3.5	106.827 ± 0.221	Post 1950 AD	Post 1950 AD
SacA 57099	4.5	-5	121.812 ± 0.241	Post 1950 AD	Post 1950 AD
SacA 57100	5.5	-5	69.402 ± 0.195	3168 - 2998	2115 - 1995
SacA 57101	6.5	-3.8	47.818 ± 0.164	6836 - 6669	5907 - 5747
SacA 57102	11.5	-2.1	47.502 ± 0.163	6897 - 6736	5985 - 5755
SacA 57103	13.5	-3	44.686 ± 0.160	7429 - 7322	6485 - 6311
SacA 57104	18.5	-4	44.244 ± 0.171	7562 - 7421	6600 - 6403

Table 1, S6 – ^{14}C results for stalagmite GN16/08 expressed as pMC percent of modern carbon and calibrated ages assuming 0 and 10% of dead carbon (DCP).

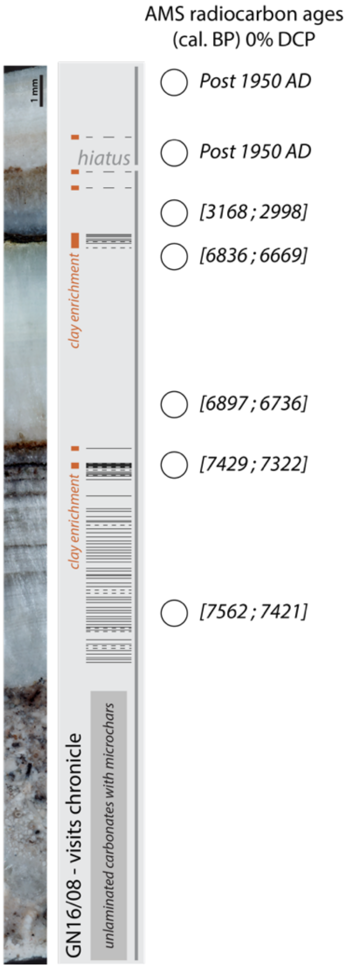

Figure 1, S6 – Section of the stalagmite with micro-levels of soot, detail of the levels and indication of the location of the $CaCO_3$ datings.

S7: Interdisciplinary analysis on soot micro-layers from stalagmite GN16/08.

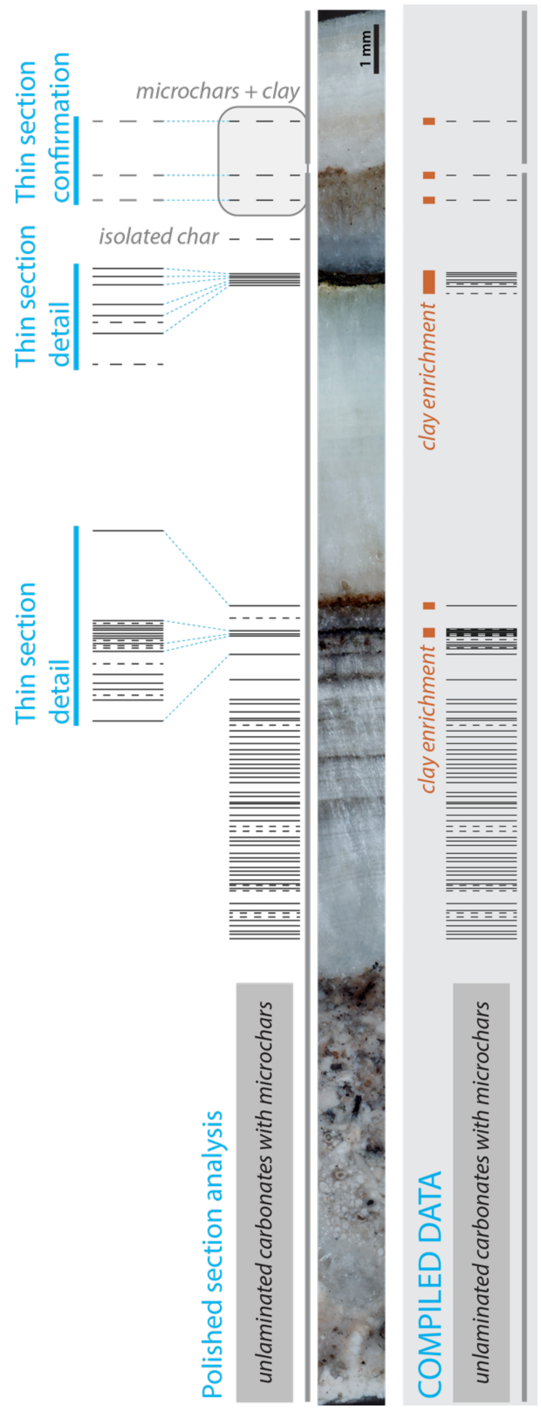

Figure 1, S7 – GN16/08. Polished section. Stitching of x50 magnification images. Reflected light. On the left side: data collected from polished section microscopic analysis and details obtained from thin section analysis (see Figure 2, S6 for correspondence between polished section and thin section observations). On the right side: Chronicle of occupations compiling all data. Data are presented as a barcode diagram, with bars representing soot films and dashed lines representing probable soot films or micro-charcoal alignments. The long vertical grey line next to the barcode represents speleothem total thickness (growth axis oriented from bottom to top).

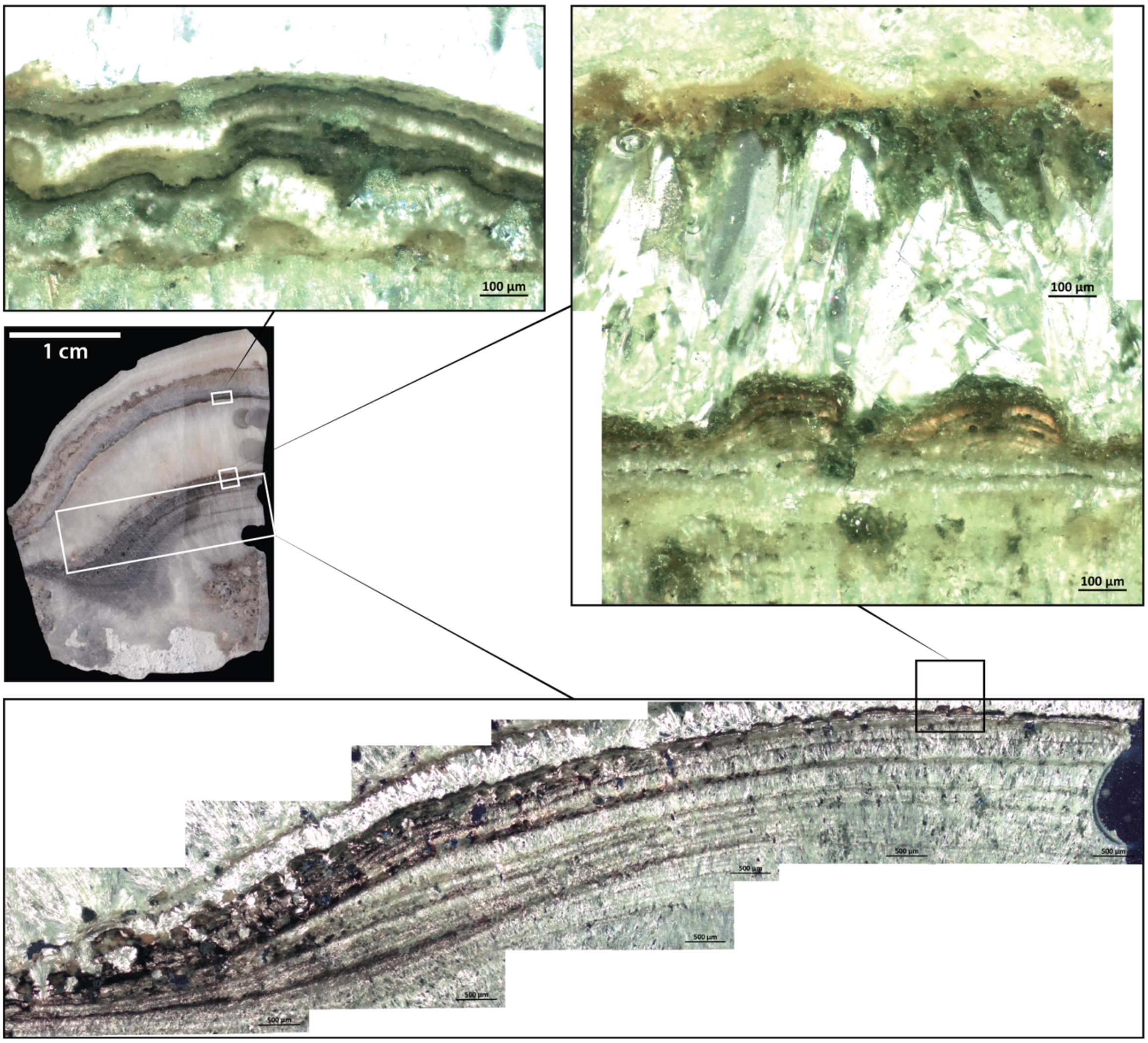

Figure 2, S7 – Polished section of sample GN16/08 and correspondence with the areas studied in thin section (in the frames). Stitching of x20 and x100 magnification images. Joint use of reflected light and crossed polarised transmitted light (RL+XPL) that makes it possible both to distinguish the soot films and to observe the carbonate matrix.

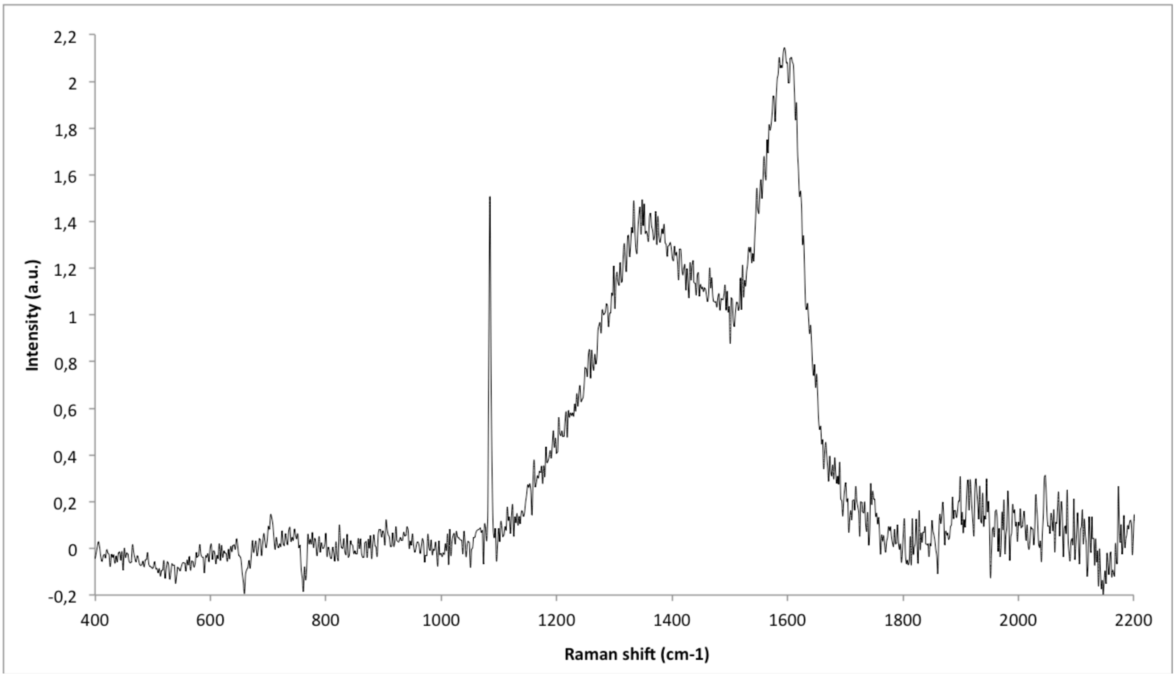

Figure 3, S7 – Raman spectrum of the black layers of stalagmite GN16/08. The peaks at 1350 and 1600 cm^{-1} are diagnostic of soot or chars and the peak at 1085 cm^{-1} is characteristic of carbonates. 514 nm laser. Power (2.5 mW). 20 s time exposure.

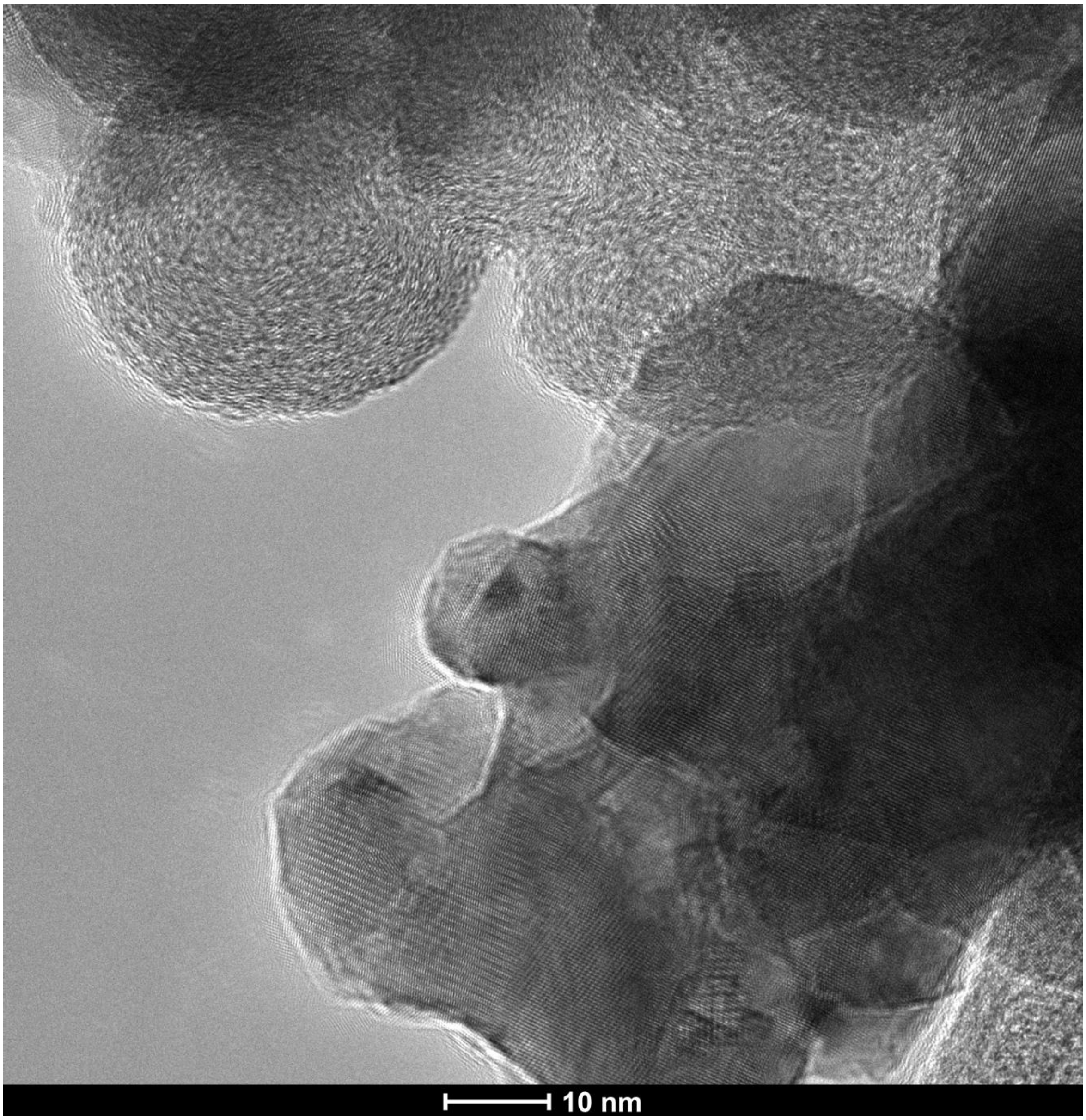

Figure 4, S7 - TEM image of spherical soot nanoparticles of the black layers of stalagmite GN16/08

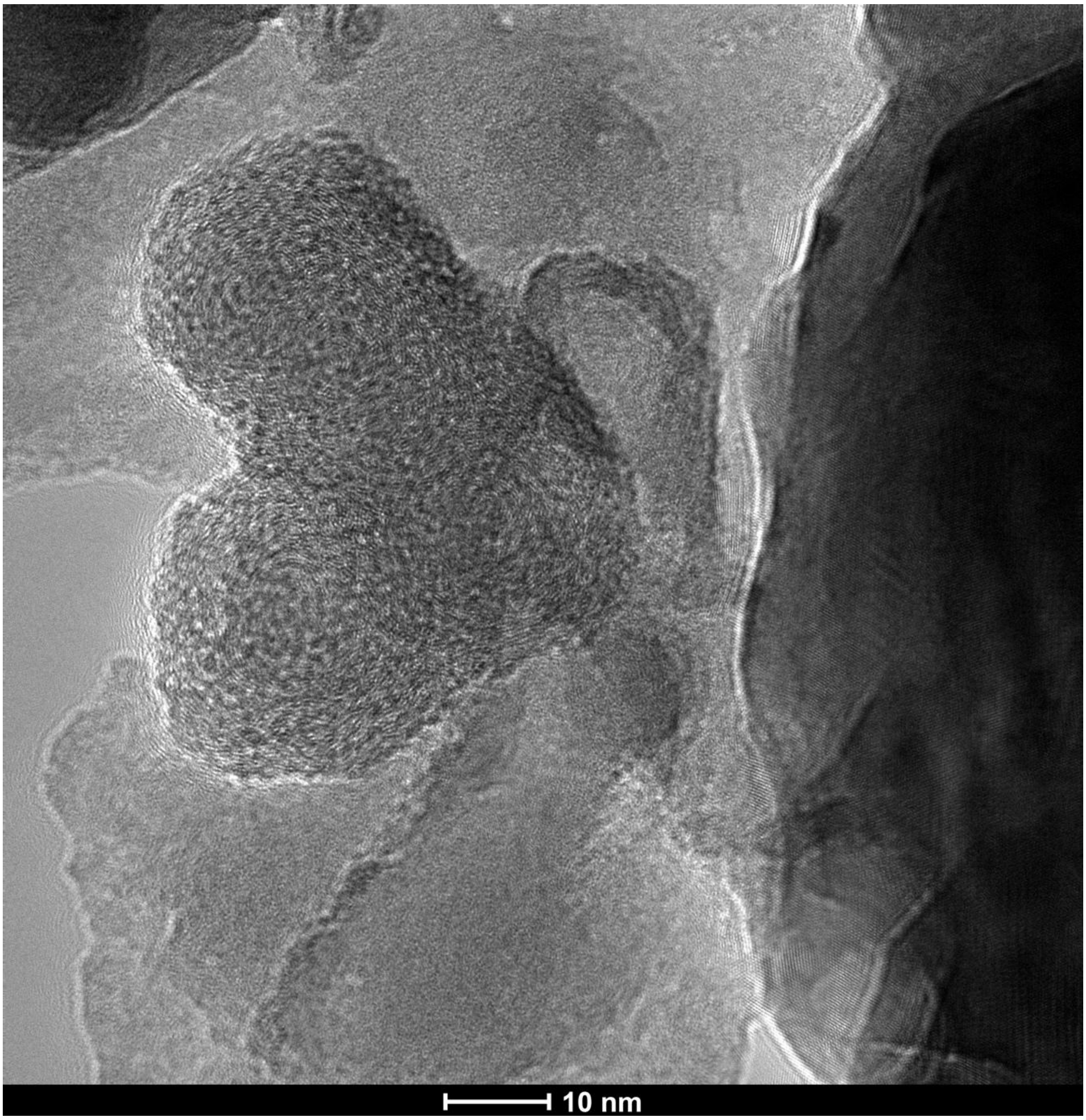

Figure 5, S7 - TEM image of spherical soot nanoparticles of the black layers of stalagmite GN16/08

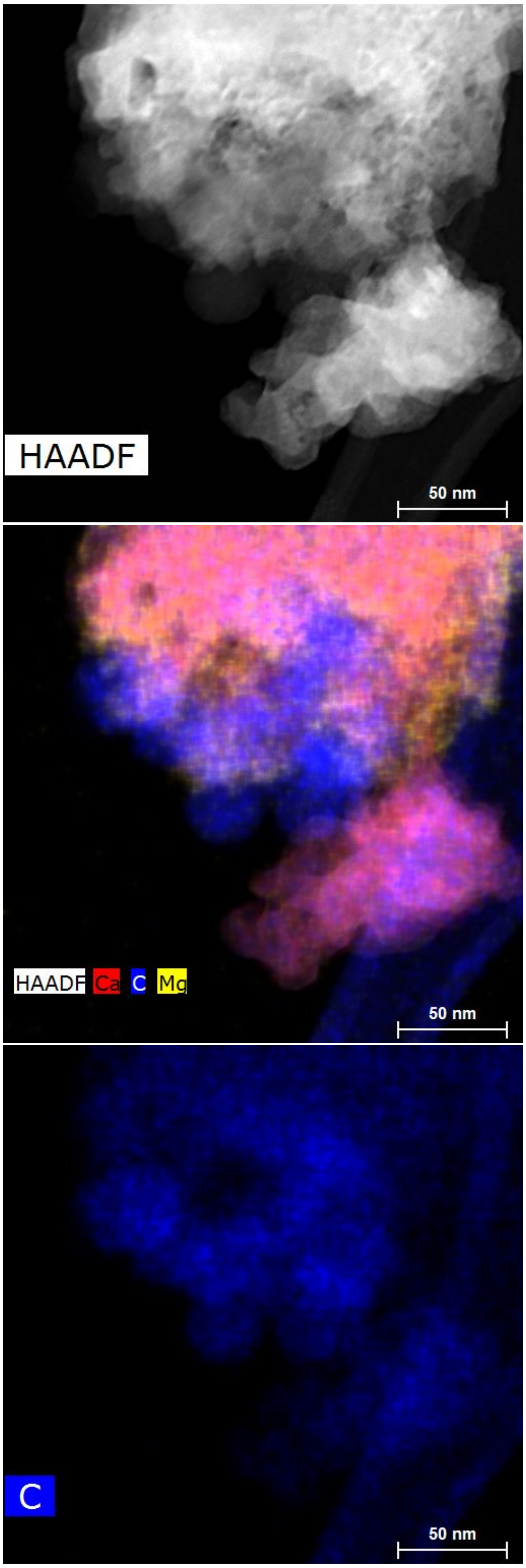

Figure 6, S7 – TEM-EDX images of spherical soot nanoparticles of the black layers of stalagmite GN16/08 (in blue the soot particles).

S8: Image of the geographic situation of the cave, with the location of the sites mentioned in the article and a photograph of the archaeological context inside the cave *(the picture shows the access to the upper galleries).*

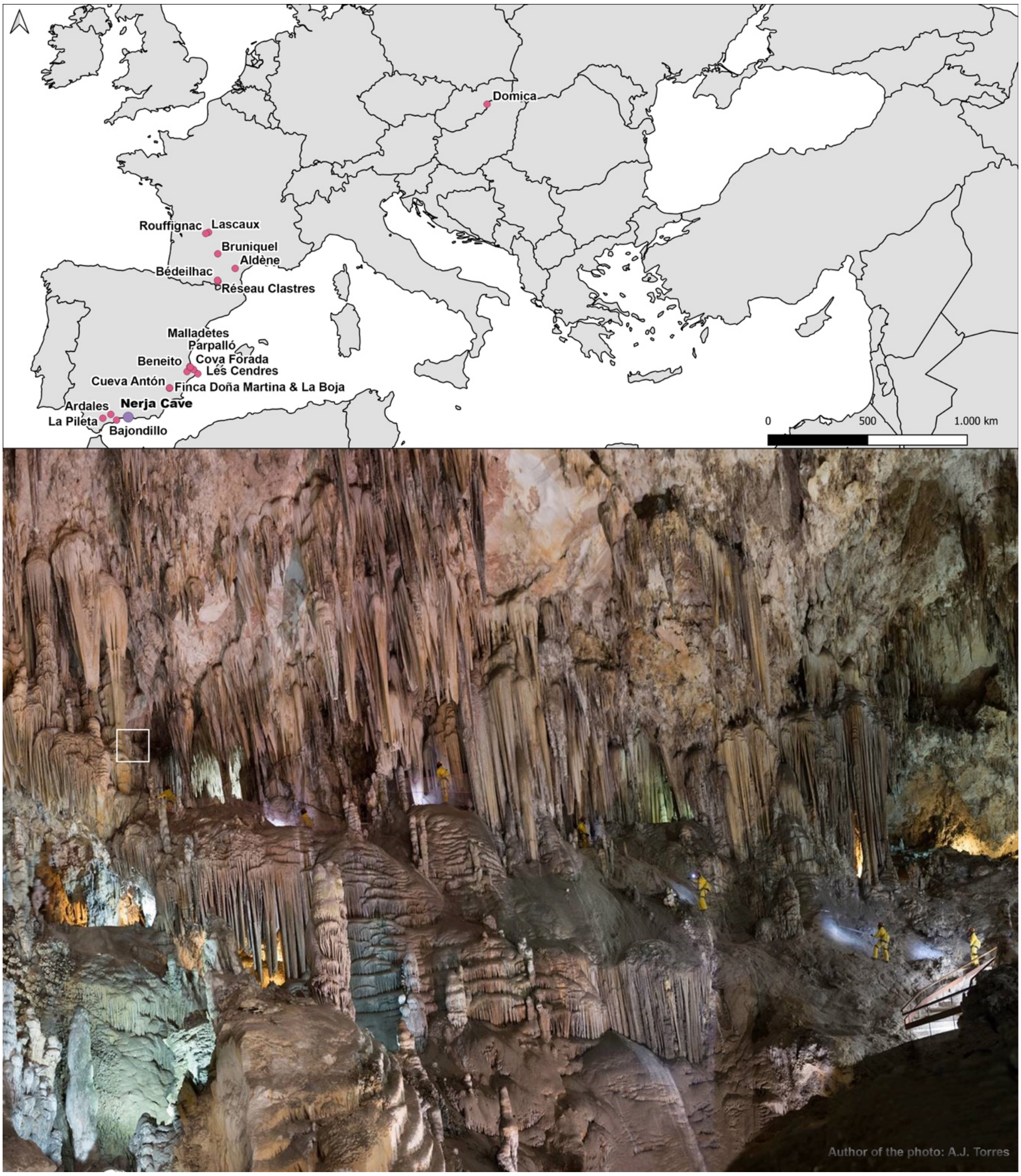

S9: References in the supplementary information.

Aguilera, M., Medina-Alcaide, M.Á, Romero, A., 2015. Nuevas aportaciones al Neolítico Antiguo de la Cueva de Nerja (Málaga, España). In V., Gonçalves, M., Diniz, A.C., Sousa, (eds.), *5º Congresso do Neolítico Peninsular*. Centro de Arqueologia da Universidade de Lisboa, Lisboa, 273-279.

Audiard, B., Blasco, T., Brossier, B., Fiorentino, G., Battipaglia, G., Théry-Parisot, I., 2018. δ 13 C referential in three Pinus species for a first archaeological application to Paleolithic contexts: "Between intra-and inter-individual variation and carbonization effect". *Journal of Archaeological Science: Reports* 20, 775-783.

Badal, E., Carrión, Y., Rivera, D., Uzquiano, P., 2003. La Arqueobotánica en cuevas y abrigos: objetivos y métodos de muestreo. In R. Buxó and R. Piqué (dirs.), *La recogida de muestras en arqueobotánica: objetivos y propuestas metodológicas*. Museu d'Arqueologia de Catalunya, Barcelona, 19-29.

Bonneau, A., Pearce, D.G., Pollard, A.M., 2012. A multi-technique characterization and provenance study of the pigments used in San rock art, South Africa. *Journal of Archaeological Science* 39, 287-294.

Bronk Ramsey, C., 1995. Radiocarbon calibration and analysis of stratigraphy: the OxCal program. *Radiocarbon* 37 (2), 425-430.

Bronk Ramsey, C., 2001. Development of the radiocarbon calibration program. *Radiocarbon* 43 (2A), 355-363.

Bronk Ramsey, C., 2006. New Approaches to Constructing Age Models: OxCal4. *PAGES News* 14(3), 14-15.

Bronk Ramsey, C., 2009a. Bayesian Analysis of Radiocarbon Date. *Radiocarbon* 51(1), 337-360.

Bronk Ramsey, C., 2009b. Dealing with outliers and offsets in radiocarbon dating. *Radiocarbon* 51 (3), 1023-1045.

Bronk Ramsey, C., Lee, S. 2013. Recent and planned developments of the program OxCal. *Radiocarbon* 55(2), 720-730.

Carrión, Y., Verdasco, C., Morales, J.V., Aura, J.E., 2018. Au-delà du radiocarbone: analyse de taxons et contexte combinés pour la détection de problèmes taphonomiques. Un exemple dans les Grottes de Santa Maira (Alicante, Espagne). *ArcheoSciences. Rev. d'archéométrie* 42, 35-43.

Cottereau, E., Arnold, M., Moreau, C., Baqué, D., Bavay, D., Caffy, I., Comby, C., Dumoulin, J.P., Hain, S., Perron, M., Salomon, J., Setti, V., 2007. Artemis. The new 14C AMS at LMC14 in Saclay. France. *Radiocarbon* 49(2), 291-299.

Deldicque, D., Rouzaud, J.N., Velde, B., 2016. A Raman-HRTEM study of the carbonization of wood: A new Raman-based paleothermometer dedicated to archaeometry. *Carbon* 102, 319-329.

Dumoulin, J.P., Comby-Zerbino, C., Delqué-Količ, E., Moreau, C., Caffy, I., Hain, S., ... & Beck, L., 2017. Status report on sample preparation protocols developed at the LMC14 Laboratory, Saclay, France: from sample collection to 14C AMS measurement. *Radiocarbon* 59(3), 713-726.

Fontugne, M., Hatté, C., Valladas, H., Tisnérat, N., Zazzo, A., Oberlin, C., Delque-Kolic, E., Moreau, C., Berthier, B., 2014. De l'utilisation des isotopes stables du carbone dans la datation par la méthode du radiocarbone. *L'Anthropologie* 118 (2), 194-200.

García, L., Guindeo, A., Peraza, C., De Palacios, P., 2003. *La madera y su anatomía*. Mundiprensa.

Jouve, G., 2013. Utilisation des isotopes stables pour l'identification de l'origine du carbone dans la datation du charbon de bois du Paléolithique. *L'Anthropologie* 117 (4), 413-419.

Martínez-Ramírez, S., Sánchez-Cortés, S., García-Ramos, J.V., Domingo, C., Fortes, C., Blanco-Varela, M.T., 2003. Micro-Raman spectroscopy applied to depth profiles of carbonates formed in lime mortar. *Cement and Concrete Research* 33, 2063-2068.

Medina-Alcaide, M.Á., Sanchidrián, J.L., Zapata, L., 2015. Lighting the dark: Wood charcoal analysis from Cueva de Nerja (Málaga, Spain) as a tool to explore the context of Palaeolithic rock art. *Comptes Rendus - Palevol* 14, 411-422.

Medina-Alcaide, M.Á., Garate, D., Intxaurbe, I., Sanchidrián, J.L., Rivero, O., Ferrier, C., Mesa, M.D., Pereña, J., Líbano, I., 2021. The conquest of the dark spaces: An experimental approach to lighting systems in Paleolithic caves. *PLOS ONE* 16, e0250497.

Mook, W.G., van der Plicht, J., 1999. Reporting 14C activities and concentrations. *Radiocarbon* 41(3), 227-239.

Moreau, C., Messager, C., Berthier, B., Hain, S., Thellier, B., Dumoulin, J.P., Caffy, I., Sieudat, M., Beck, L., 2020. ARTEMIS, the 14C AMS facility of the LMC14 National Laboratory: a status report on quality control and microsample procedures. *Radiocarbon* 62, 1755-1770.

Morrell, B., 2019. *La cronología como medio de interpretación social: los contextos funerarios del NE de la península ibérica entre finales del V inicios del IV milenio cal bc.* Doctoral dissertation, Universitat Autònoma de Barcelona.

Pawlyta, M., Hercman, H., 2016. Transmission electron microscopy (TEM) as a tool for identification of combustion products: application to black layers in speleothems. *Annales Societatis Geologorum Poloniae* 86(2), 237-248.

Pons-Branchu, E., Sanchidrián, J. L., Fontugne, M., Medina-Alcaide, M. Á., Quiles, A., Thil, F., & Valladas, H. 2020. U-series dating at Nerja cave reveal open system. Questioning the Neanderthal origin of Spanish rock art. *Journal of Archaeological Science* 117, 10.1016/j.jas.2020.105120

Pons-Branchu, E., Barbarand, J., Caffy, I., Dapoigny, A., Drugat, L., Dumoulin, J.P., Medina-Alcaide, M.Á., Nouet, J., Sanchidrián, J.L., Tisnérat, N., Jiménez de Cisneros, C., Valladas, H., 2022. U-series and radiocarbon cross dating of speleothems from Nerja Cave (Spain): Evidence of open system behavior. Implication for the Spanish rock art chronology. *Quaternary Science Reviews* 290, 10.1016/j.quascirev.2022.107634

Quiles, A., Valladas, H., Geneste, J.M., 2014. Datation 14C: méthode, calibration et modélisation, de nouvelles perspectives? Contribution à la chronologie de l'art pariétal au Paléolithique supérieur. *Paléo Rev. d'archéologie préhistorique* nº Extra 2014, 27-40.

Reimer, P.J., Austin, W.E.N., Bard, E., Bayliss, A., Blackwell, P.G., Ramsey, C.B., Butzin, M., Cheng, H., Edwards, R.L., Friedrich, M., Grootes, P.M., Guilderson, T.P., Hajdas, I., Heaton, T.J., Hogg, A.G., Hughen, K.A., Kromer, B., Manning, S.W., Muscheler, R., Palmer, J.G., Pearson, C., van der Plicht, J., Reimer, R.W., Richards, D.A., Scott, E.M., Southon, J.R., Turney, C.S.M., Wacker, L., Adophi, F., Büntgen, U., Capano, M., Fahrni, S., Fogtmann-Schulz, A., Friedrich, R., K€ohler, P., Kudsk, S., Miyake, F., Olsen, J., Reinig, F., Sakamoto, M., Sookdeo, A., Talamo, S., 2020. The IntCal20 Northern Hemisphere radiocarbon calibration curve (0-55kcal BP). *Radiocarbon* 62, 725-757.

Romero, A., Cristo, A., Medina-Alcaide, M.Á., Sanchidrián, J.L., 2012. Datación del contexto arqueológico y frecuentación pleistocena en la Cueva de Nerja (Málaga, España). *Préhistoire, art et sociétés: Bulletin de la Société Préhistorique de l'Ariège* 65-66, 1105-1122.

Sadezky, A., Muckenhuber, H., Grothe, H., Niessner, R., Pöschl, U., 2005. Raman microspectroscopy of soot and related carbonaceous materials: Spectral analysis and sample preparation protocols developed at the LMC14 Laboratory, Saclay, France: from sample collection to 14C AMS measurement. *Radiocarbon* 59(3), 713-726.

Sanchidrián, J.L., Márquez, A., Valladas, H., Tisnerat, N., 2001. Direct dates for Andalusian Rock Art (Spain). *I.N.O.R.A.* 29, 15-19.

Sanchidrián, J.L., Valladas, H., Medina-Alcaide, M.Á., Pons-Branchu, E., Quiles, A., 2017. New perspectives for 14C dating of parietal markings using CaCO3 thin layers: An example in Nerja cave (Spain). *Journal of Archaeological Science: Reports* 12, 74-80.

Schweingruber, F.H., 1990. *Anatomy of European woods. Anatomy of European woods.* Paul Haupt.

Tisnerat, N., Poupeau, J.J., Tannau, J.F., Paterne, M., 2001. Development of a semi-automated system for routine preparation of carbonate samples. *Radiocarbon* 43(2A), 299-304.

Valladas, H., Pons-Branchu, E., Dumoulin, J.P., Quiles, A., Medina-Alcaide, M.Á., Sanchidrian, J.L., 2017. U/Th and C-14 cross dating of parietal calcite deposits: application to Nerja cave (Andalusia, Spain) and future perspectives. *Radiocarbon* 59(6), 1955-1967.

Valladas, H., Tisnerat, N., Cachier, H., Arnold, M., 1999. Datation directe des peintures prehistoriques par la methode du carbone 14 en spectrometrie de masse par accelerateur. *Mémoires la Société préhistorique française* 26, 39-44.

Vandevelde, S., 2021. Les rythmicités d'occupation d'un site au sein d'un territoire. Approche diachronique. In L, S., Y, G., L, M., P, Y. (Eds.), Mandrin. *Des Derniers Néandertaliens Aux Premiers Hommes Modernes En France Méditerranéenne*. A&t 4. MMSH, 694-709.

Vandevelde, S., Brochier, J.É., Desachy, B., Petit, C., Slimak, L., 2018. Sooted concretions: A new Micro-chronological Tool for High Temporal Resolution Archaeology. *Quaternary International* 474, 103-118.

Vandevelde, S., Brochier, J.É., Petit, C., Slimak, L., 2017. Establishment of occupation chronicles in Grotte Mandrin using sooted concretions: rethinking the Middle to Upper Paleolithic transition. *Journal of Human Evolution* 112, 70-78.

Vandevelde, S., Genty, D., Brochier, J.É., Petit, C., Slimak, L., 2020. Des concrétions fuligineuses en contextes archéologiques : quel potentiel informatif ?. *Géomorphologie : relief, processus, environnement* 26, 241-254.

Vernet, J.L., Bazile, E., Évin, J., 1979. Coordination des analyses anthracologiques et des datations absolues sur charbon de bois. *Bulletin de la Société préhistorique française* 76, 76-79.

Vernet, J.L., Ogereau, P., Figueral, I., Machado, C., Uzquiano, P., 2001. *Guide d´identification des charbons de bois préhistoriques et récents. Sud-Ouest de l´Europe: France, Péninsule ibérique et Îles Canaries.* C.N.R.S., Paris.
